# Supplementary figures and images for: Uncoupling the TFIIH Core and Kinase Modules leads to misregulated RNA polymerase II CTD Serine 5 phosphorylation
Source: eLife. 2026 Jun 8;15:RP110091. doi: 10.7554/eLife.110091 (PMC13246002; doi:10.7554/eLife.110091)

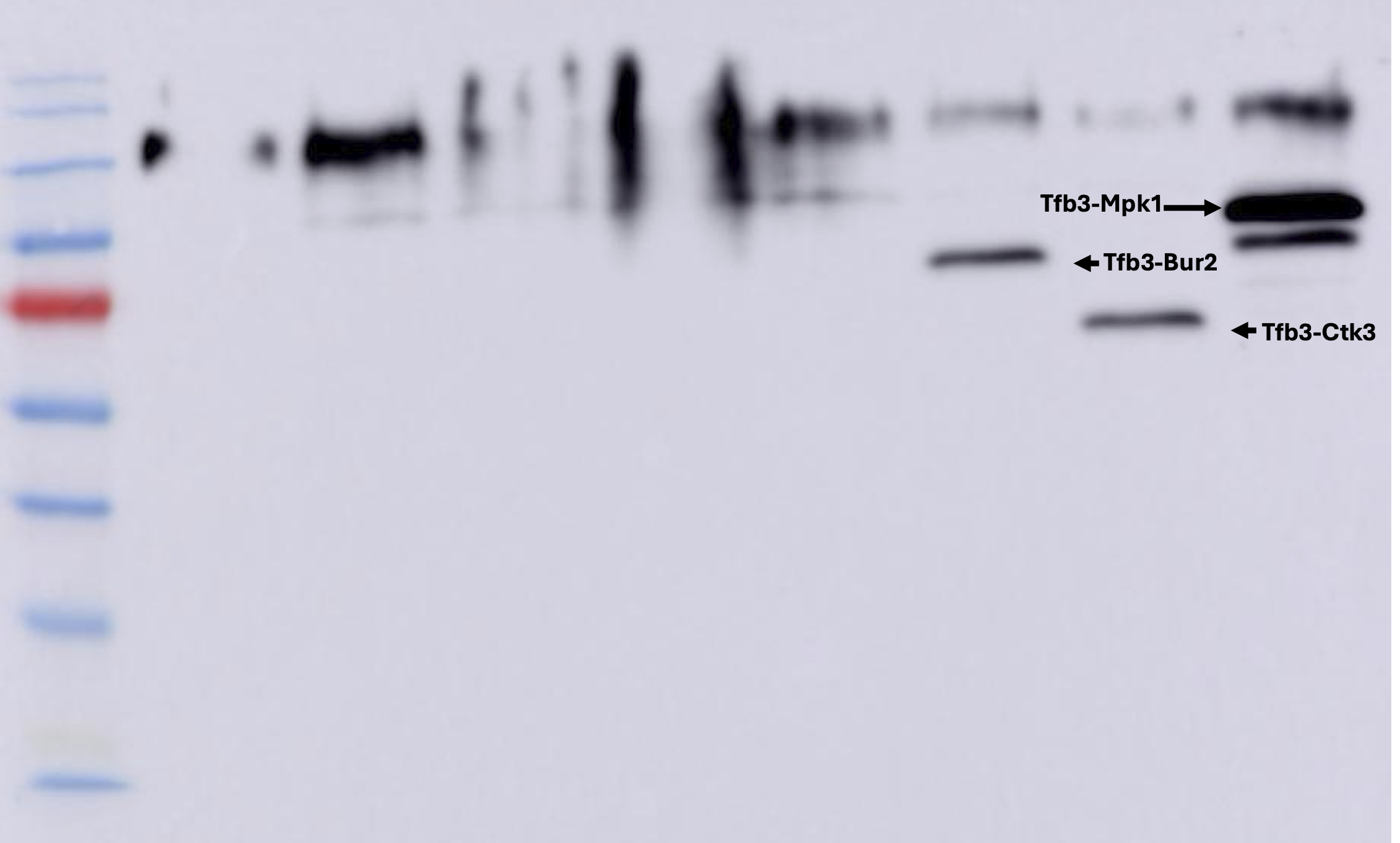

Supplement: Figure 1—figure supplement 1—source data 1. [file elife-110091-fig1-figsupp1-data1.zip › Figure 1 - Supplement 1 -source data - labeled/Figure 1 - Figure Supplement 1 - Source Data 1 .tiff]

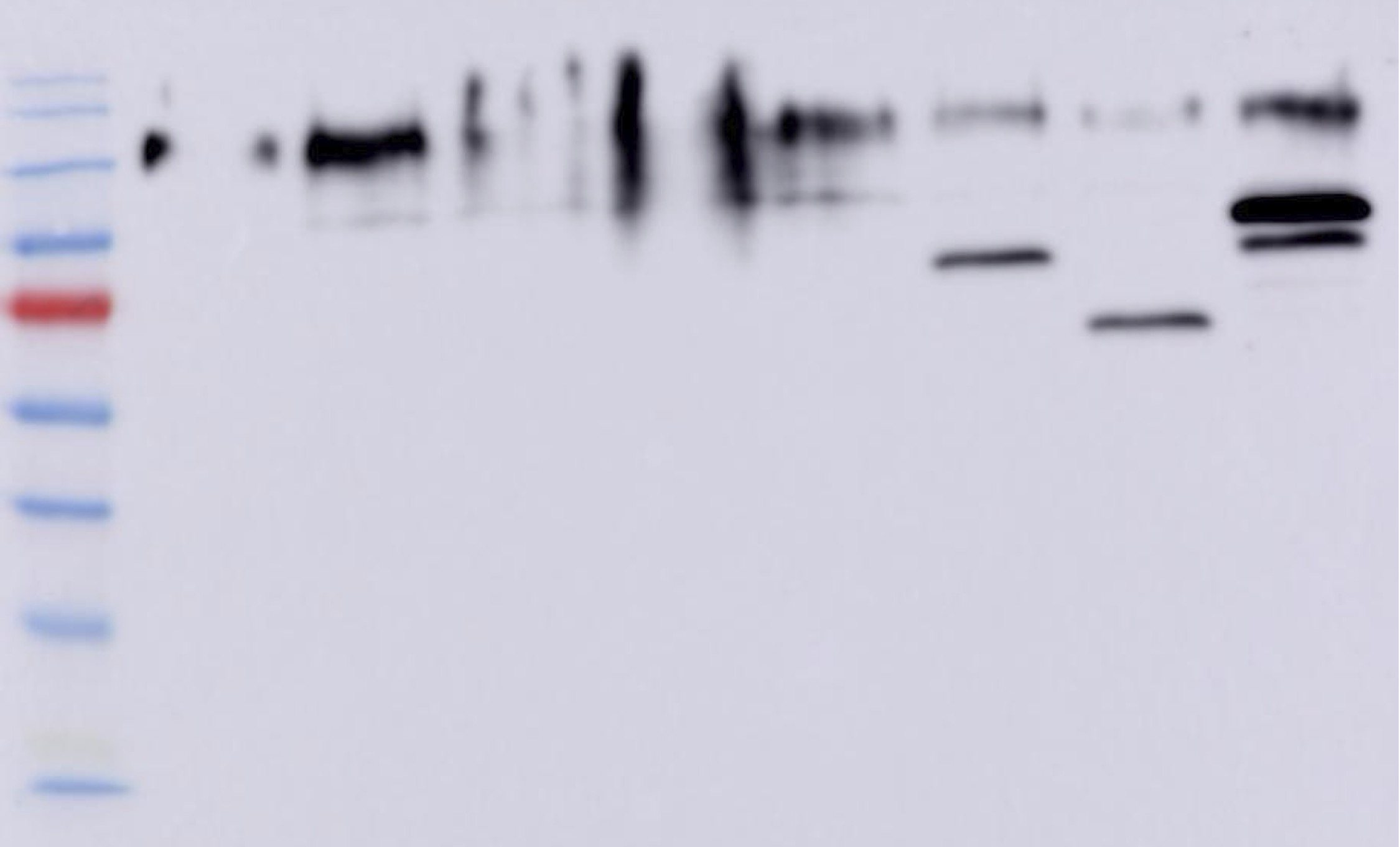

Supplement: Figure 1—figure supplement 1—source data 2. [file elife-110091-fig1-figsupp1-data2.zip › Figure 1 - Supplement 1 -source data/Figure 1 - Figure Supplement 1 - Source Data 2 .tiff]

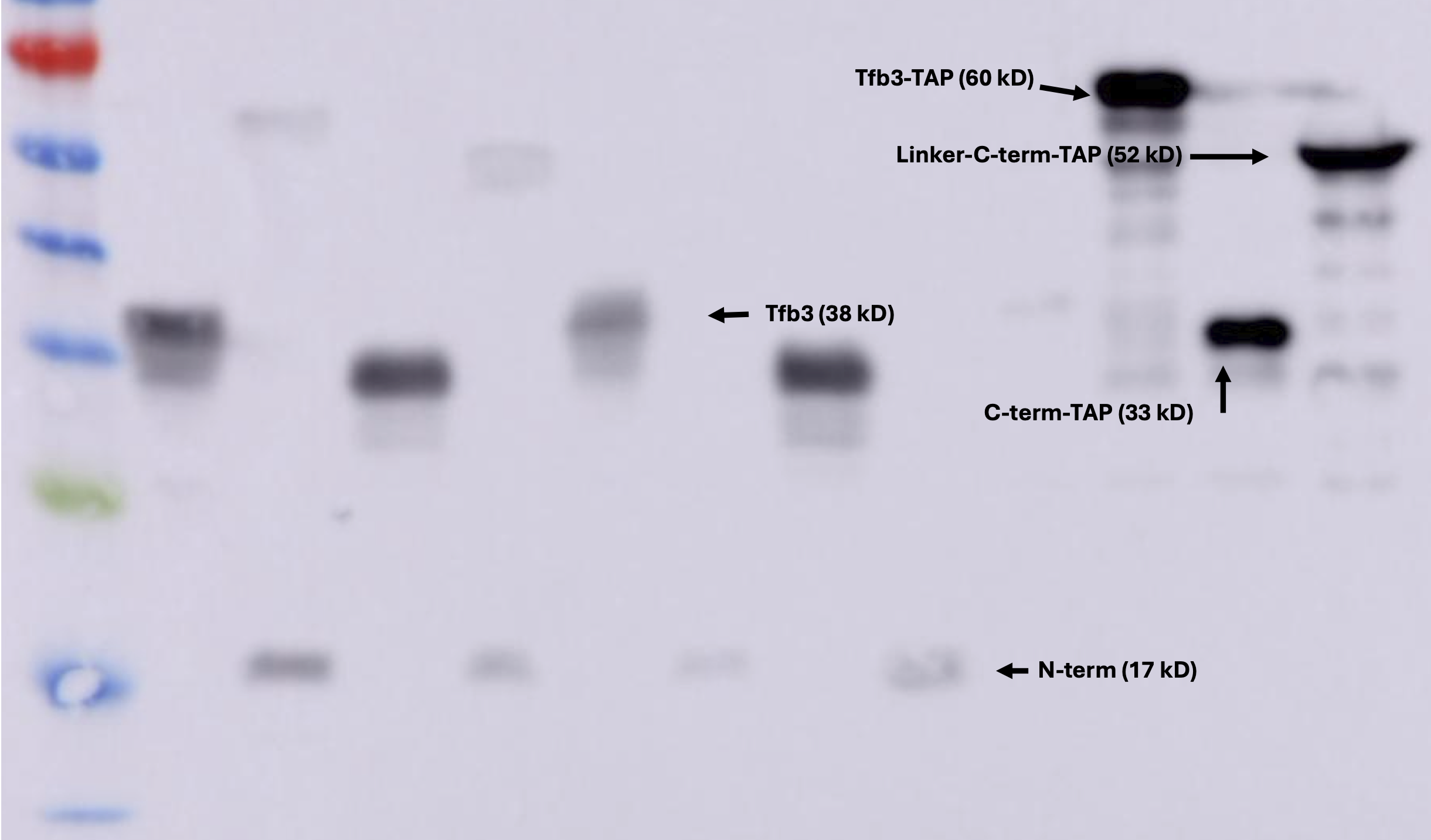

Supplement: Figure 3—source data 1. [file elife-110091-fig3-data1.zip › Figure 3 - Source Data 1 .tiff]

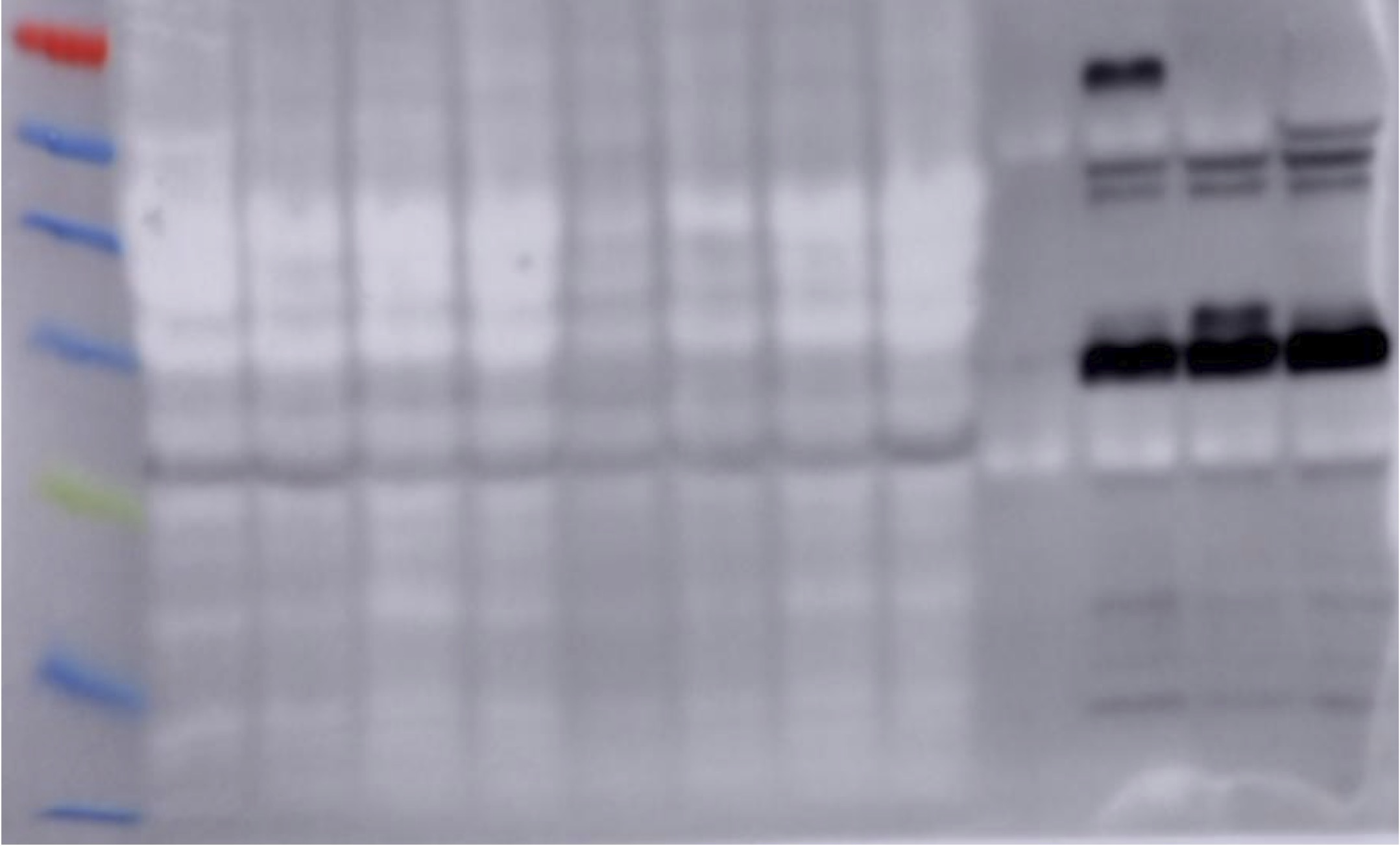

Supplement: Figure 3—source data 2. [file elife-110091-fig3-data2.zip › Figure 3 - Source Data 2 .tiff]

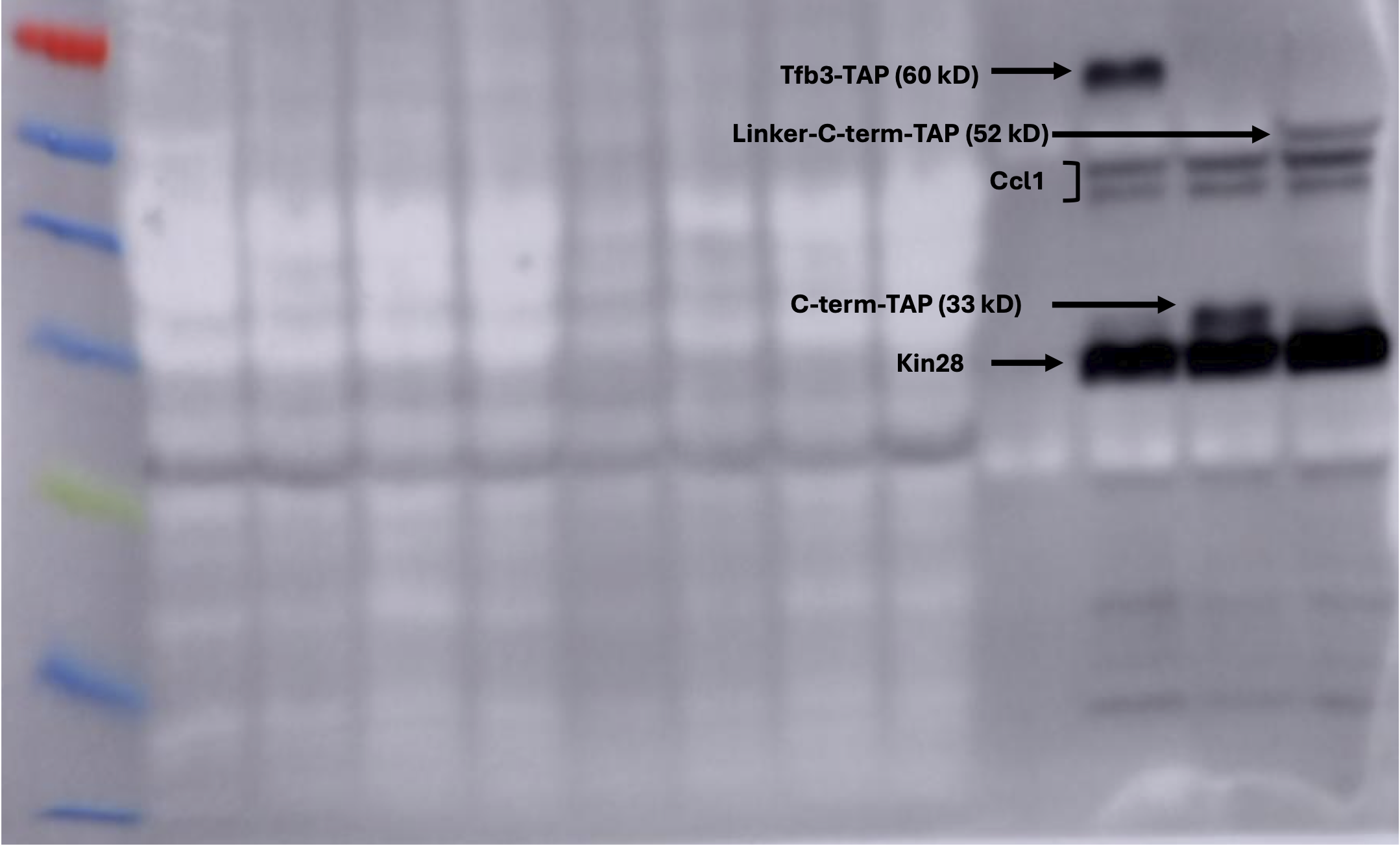

Supplement: Figure 3—source data 3. [file elife-110091-fig3-data3.zip › Figure 3 - Source Data 3 .tiff]

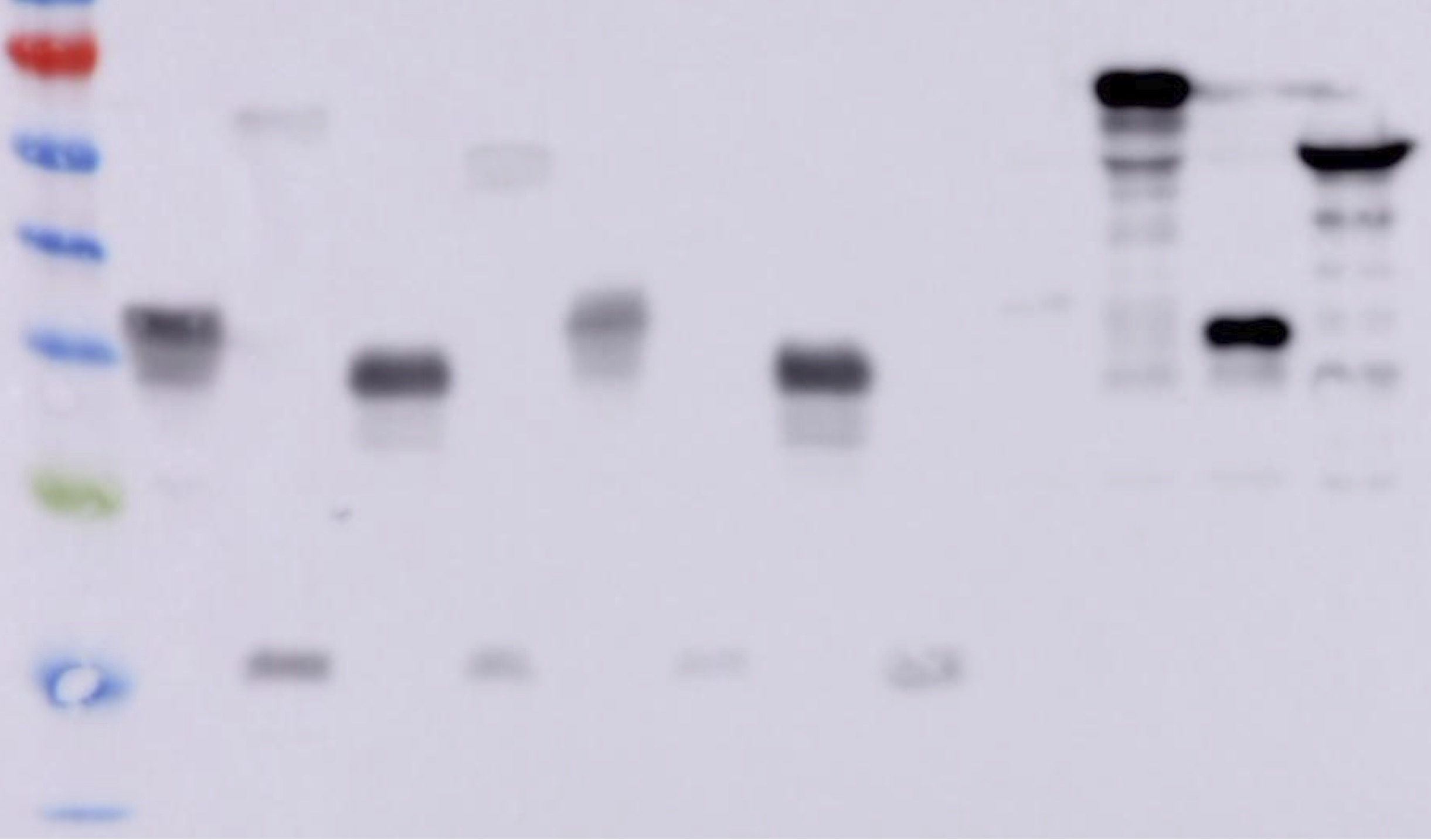

Supplement: Figure 3—source data 4. [file elife-110091-fig3-data4.zip › Figure 3 - Source Data 4 .tiff]

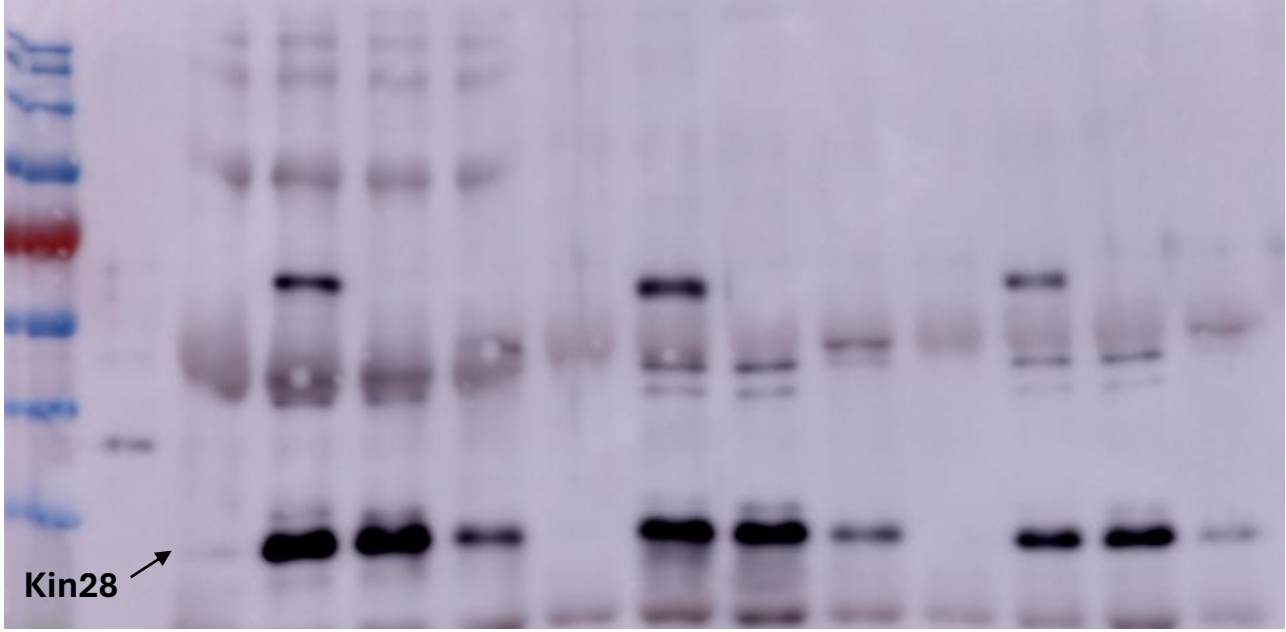

**Kin28** →

Supplement: Figure 3—figure supplement 1—source data 1. [file elife-110091-fig3-figsupp1-data1.zip › Figure 3 - Figure Supplement 1 - Source Data 1 .pdf]

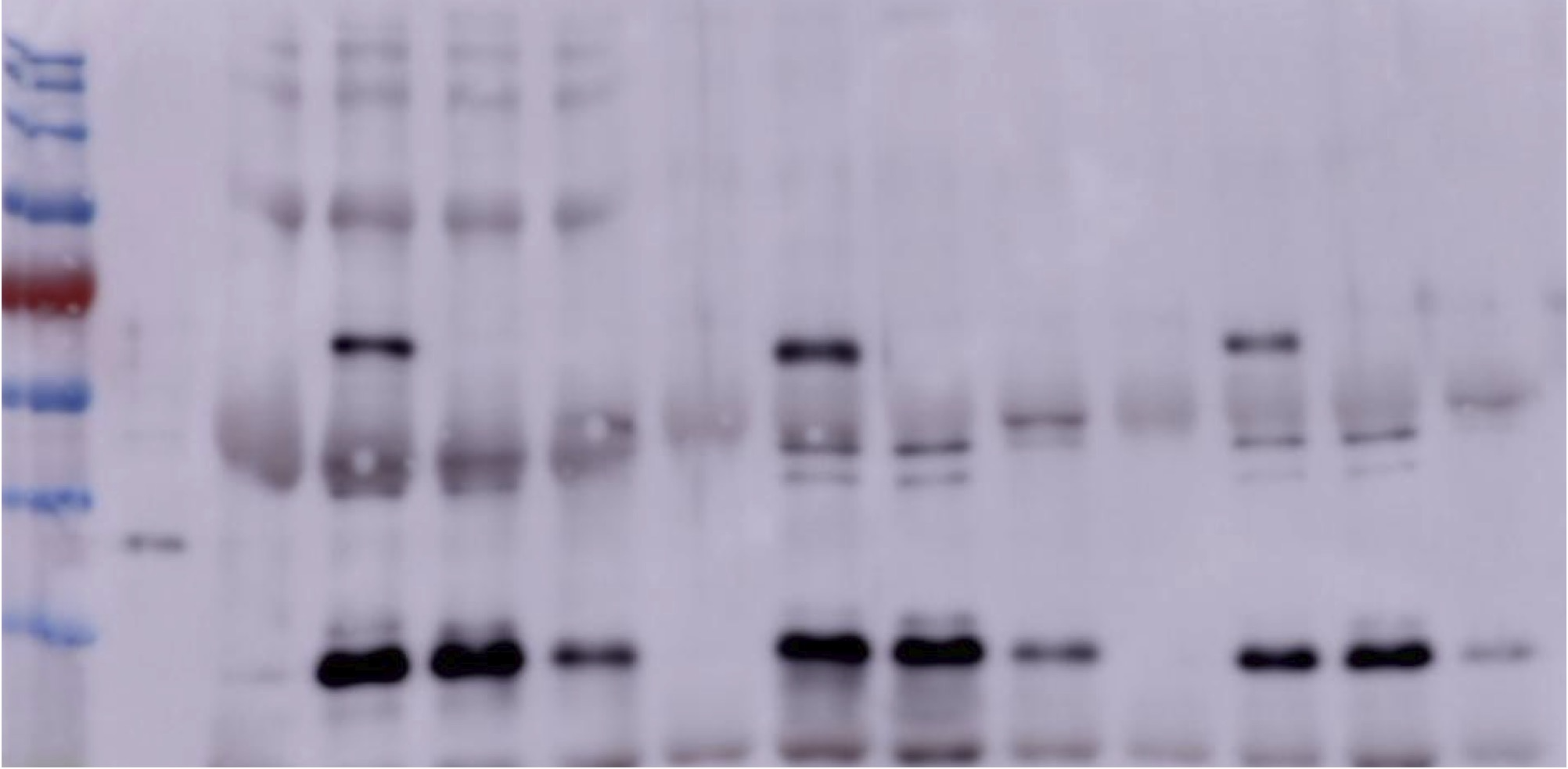

Supplement: Figure 3—figure supplement 1—source data 2. [file elife-110091-fig3-figsupp1-data2.zip › Figure 3 - Figure Supplement 1 - Source Data 2 .tiff]

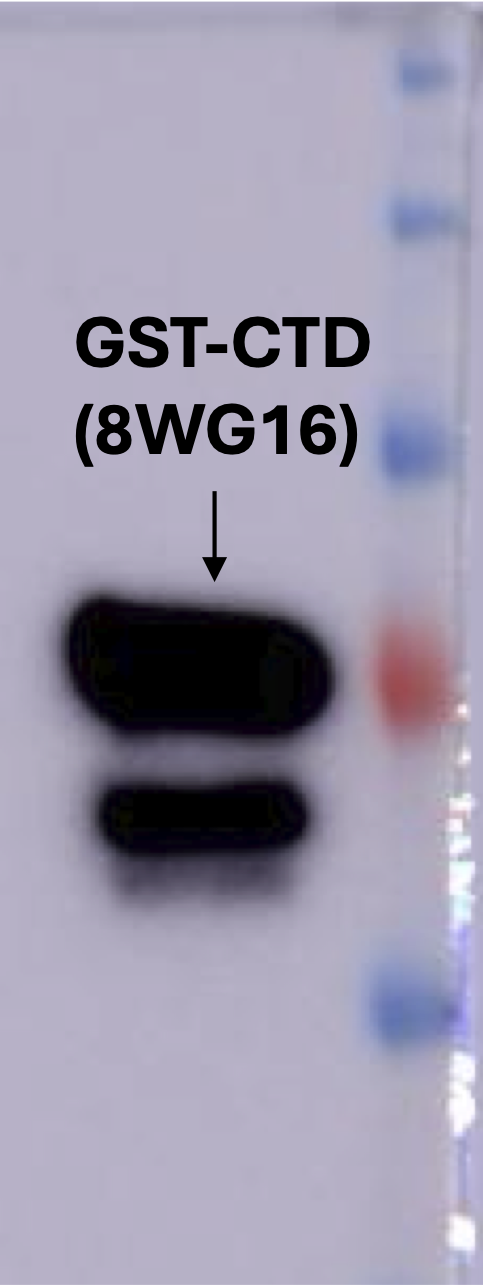

Supplement: Figure 3—figure supplement 1—source data 3. [file elife-110091-fig3-figsupp1-data3.zip › Figure 3 - Figure Supplement 1 - Source Data 3.tiff]

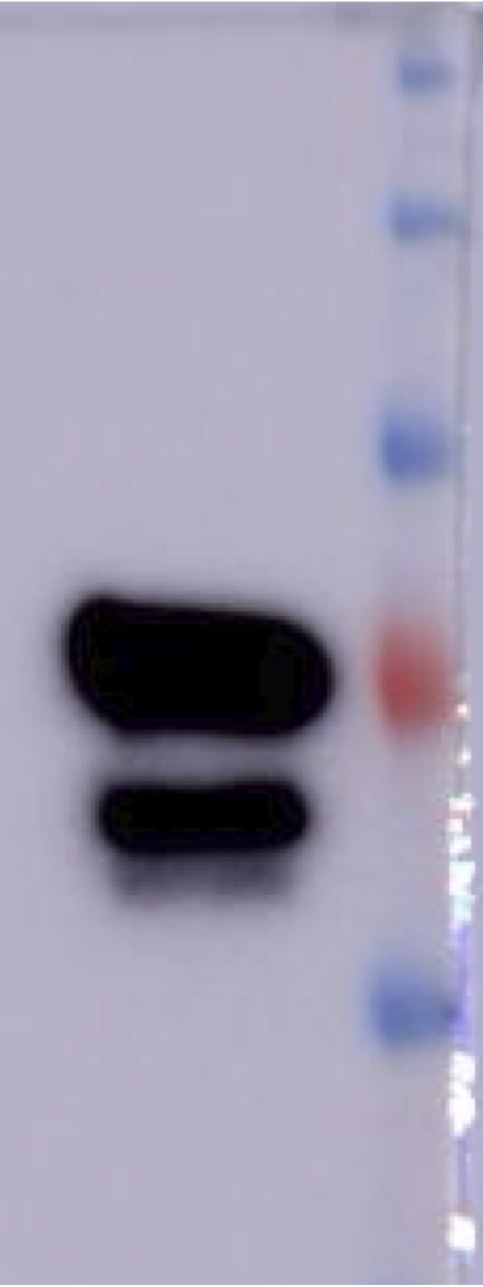

Supplement: Figure 3—figure supplement 1—source data 4. [file elife-110091-fig3-figsupp1-data4.zip › Figure 3 - Figure Supplement 1 - Source Data 4 .tiff]

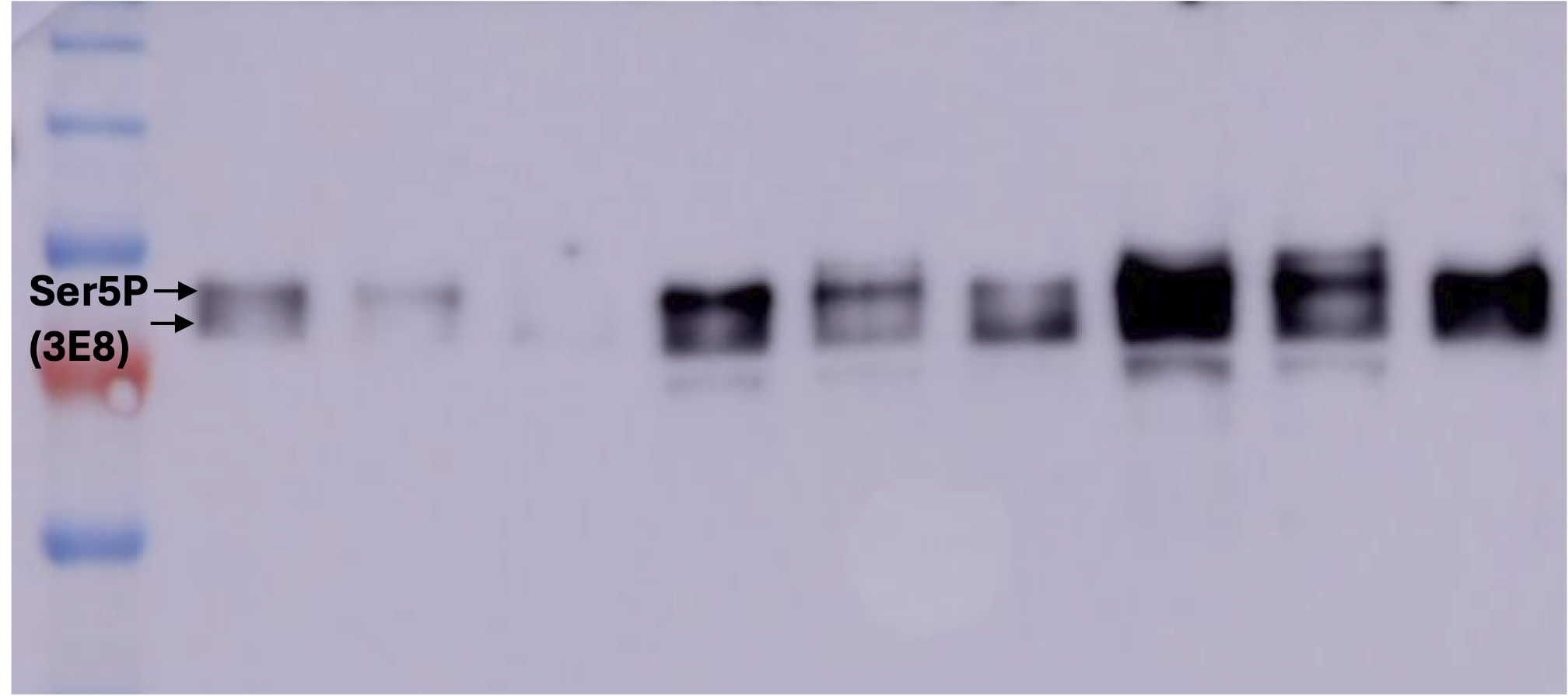

Supplement: Figure 3—figure supplement 1—source data 5. [file elife-110091-fig3-figsupp1-data5.zip › Figure 3 - Figure Supplement 1 - Source Data 5.tiff]

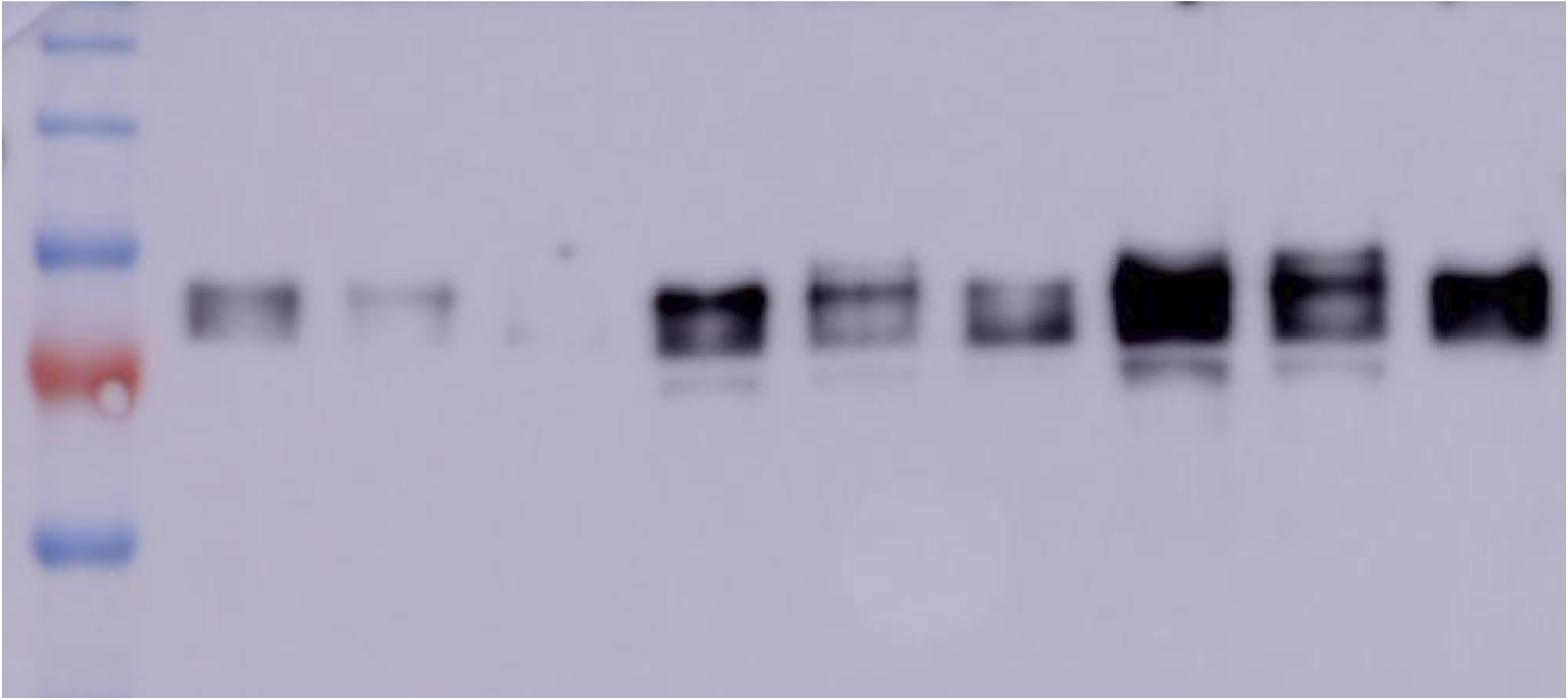

Supplement: Figure 3—figure supplement 1—source data 6. [file elife-110091-fig3-figsupp1-data6.zip › Figure 3 - Figure Supplement 1 - Source Data 6.tiff]

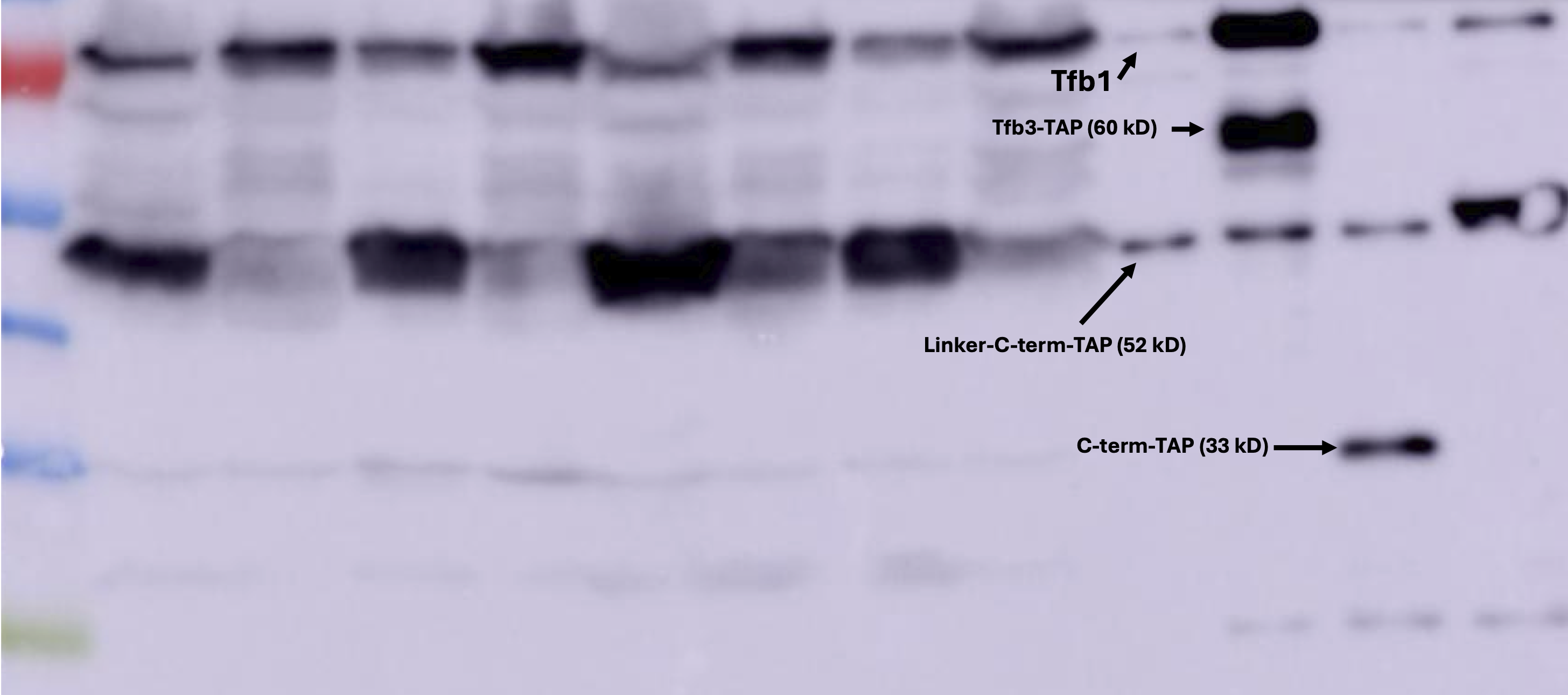

Supplement: Figure 4—source data 1. [file elife-110091-fig4-data1.zip › Figure 4 - Source Data 1 .tiff]

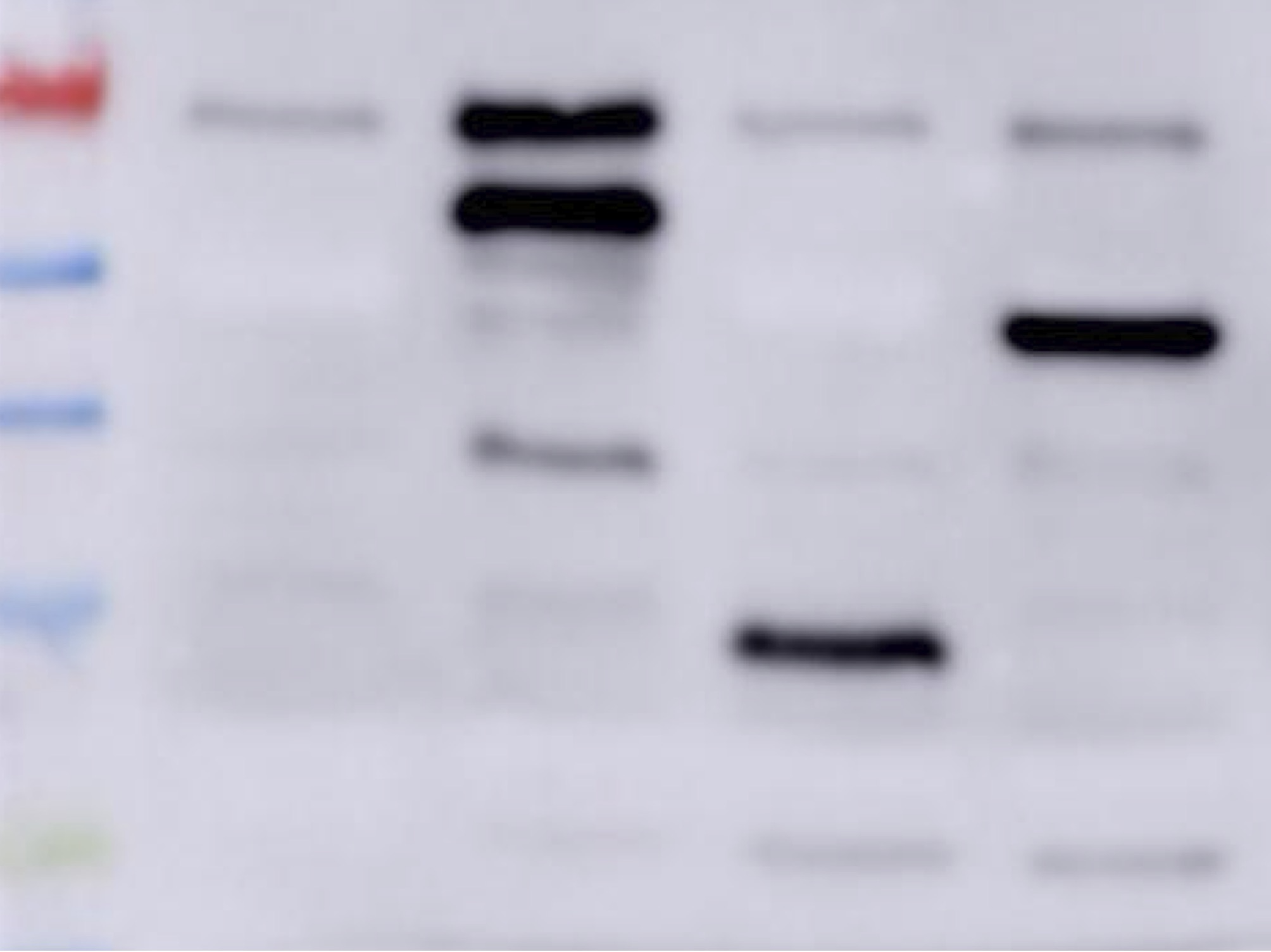

Supplement: Figure 4—source data 2. [file elife-110091-fig4-data2.zip › Figure 4 - Source Data 2 .tiff]

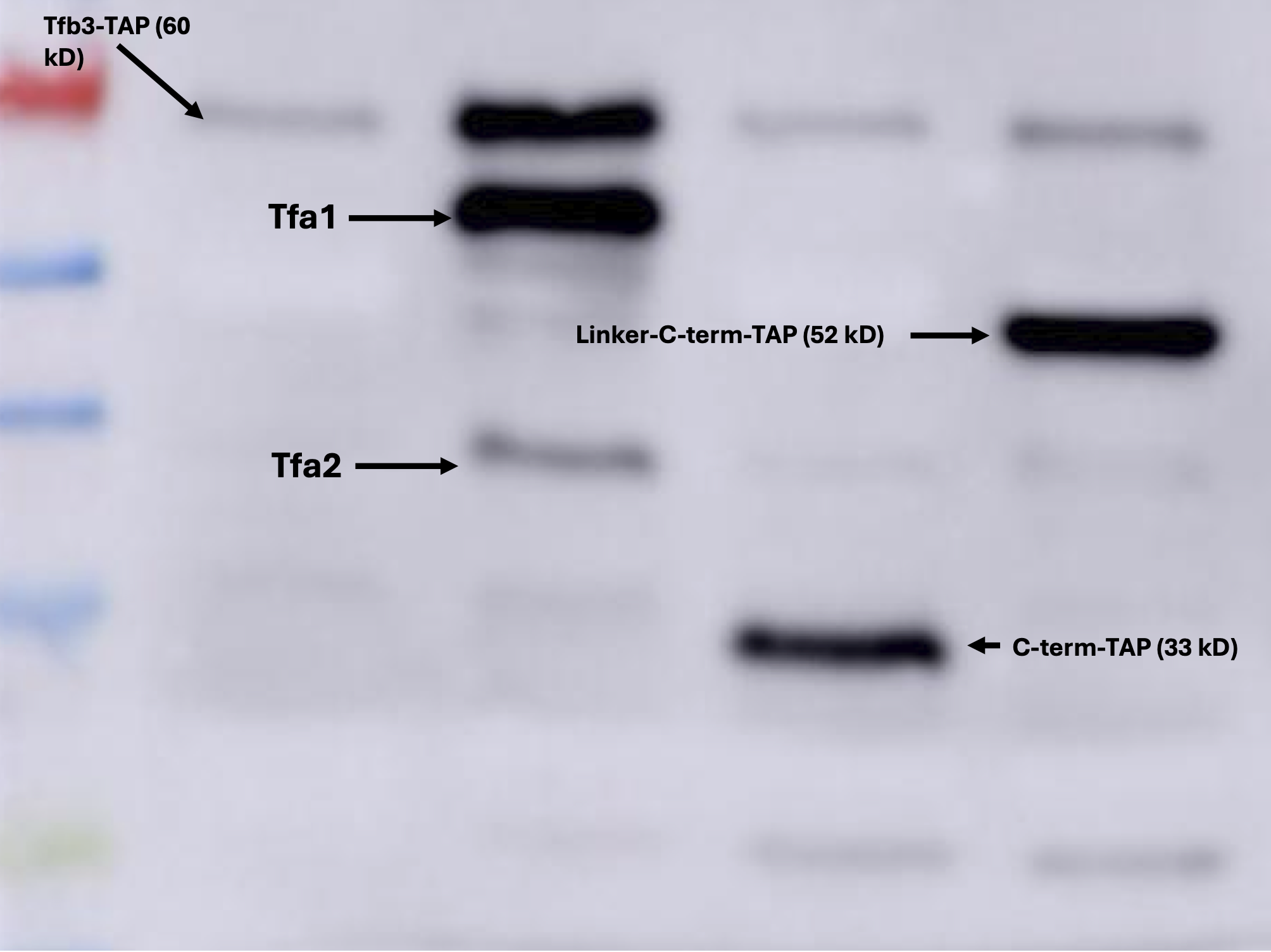

Supplement: Figure 4—source data 3. [file elife-110091-fig4-data3.zip › Figure 4 - Source Data 3 .tiff]

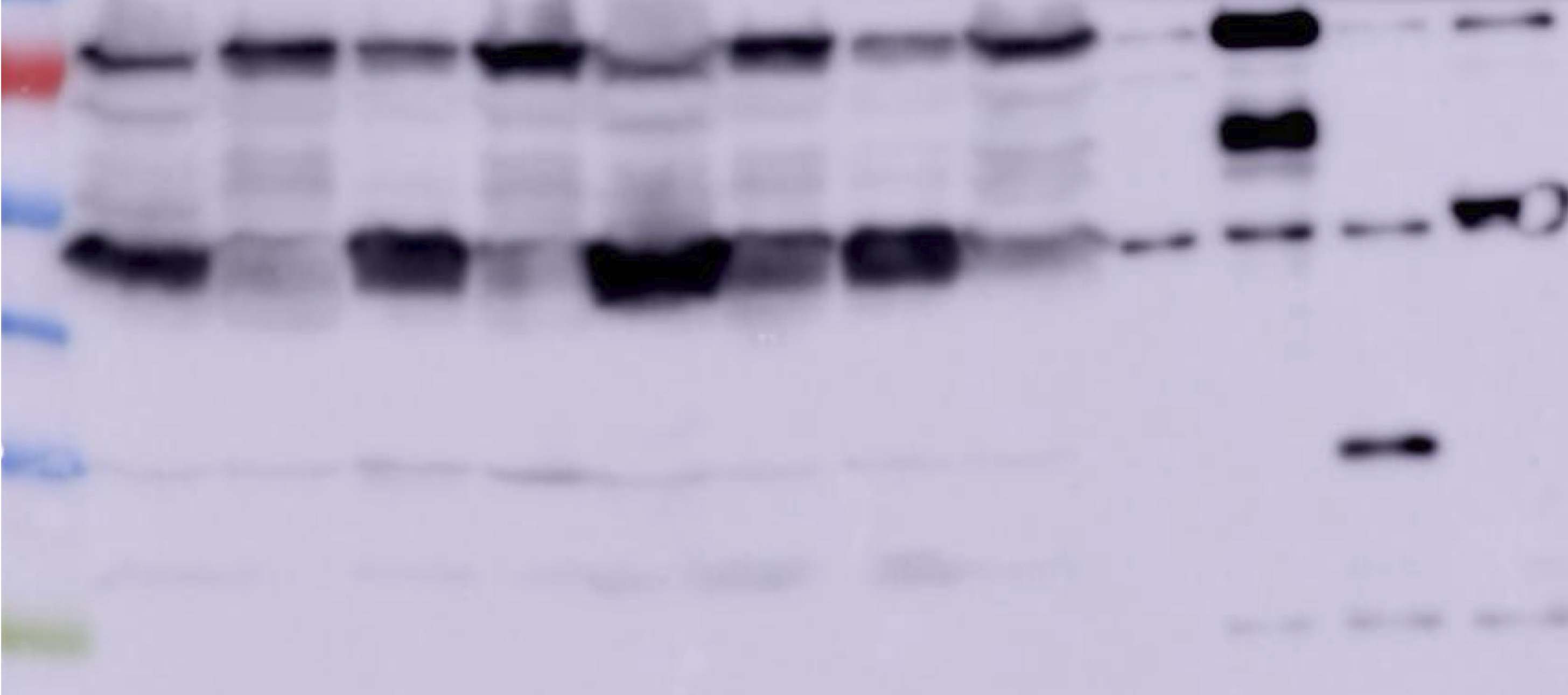

Supplement: Figure 4—source data 4. [file elife-110091-fig4-data4.zip › Figure 4 - Source Data 4 .tiff]

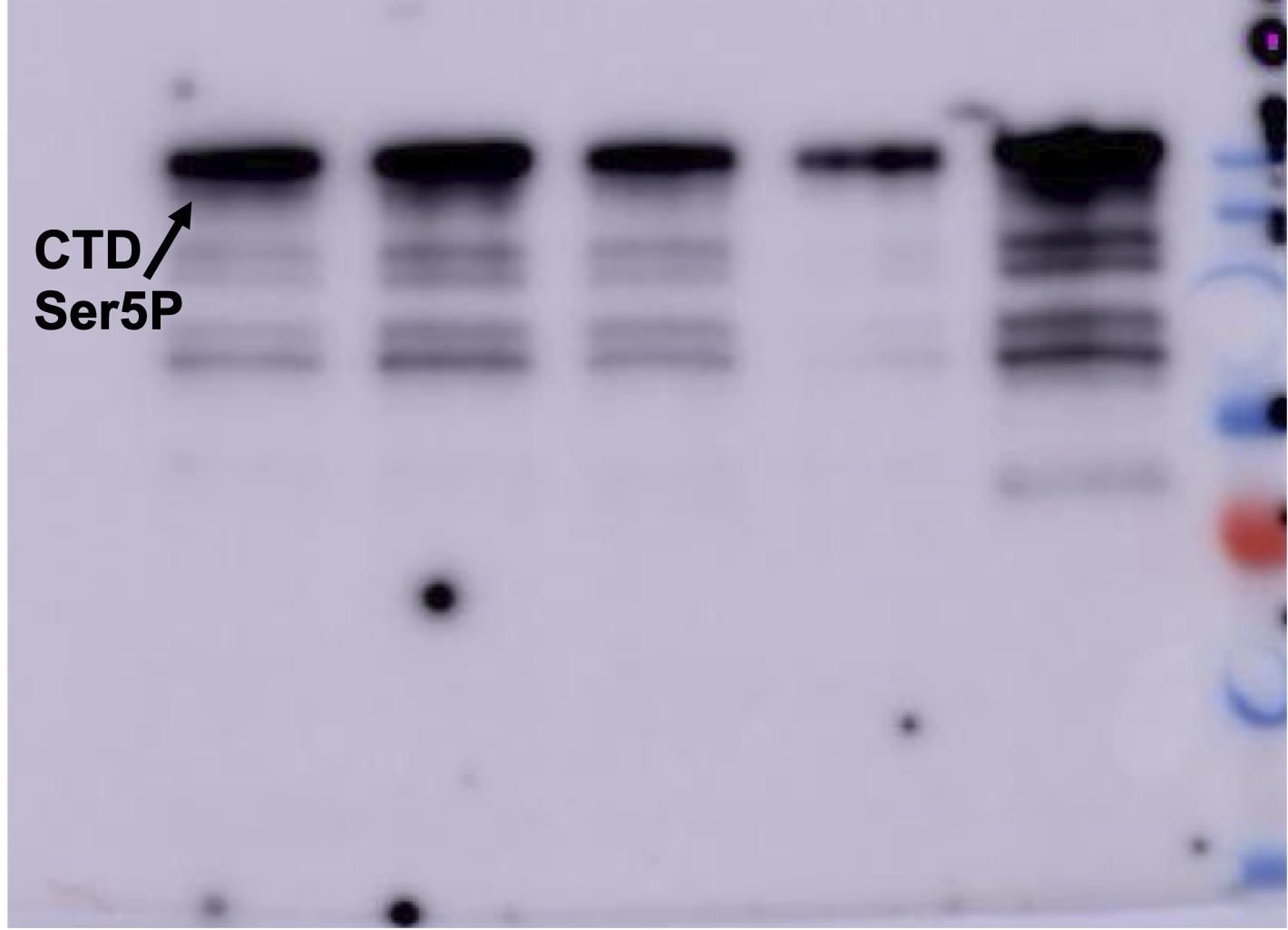

Supplement: Figure 5—figure supplement 2—source data 1. [file elife-110091-fig5-figsupp2-data1.zip › Figure 5 - Figure Supplement 2 - Source Data 1.tiff]

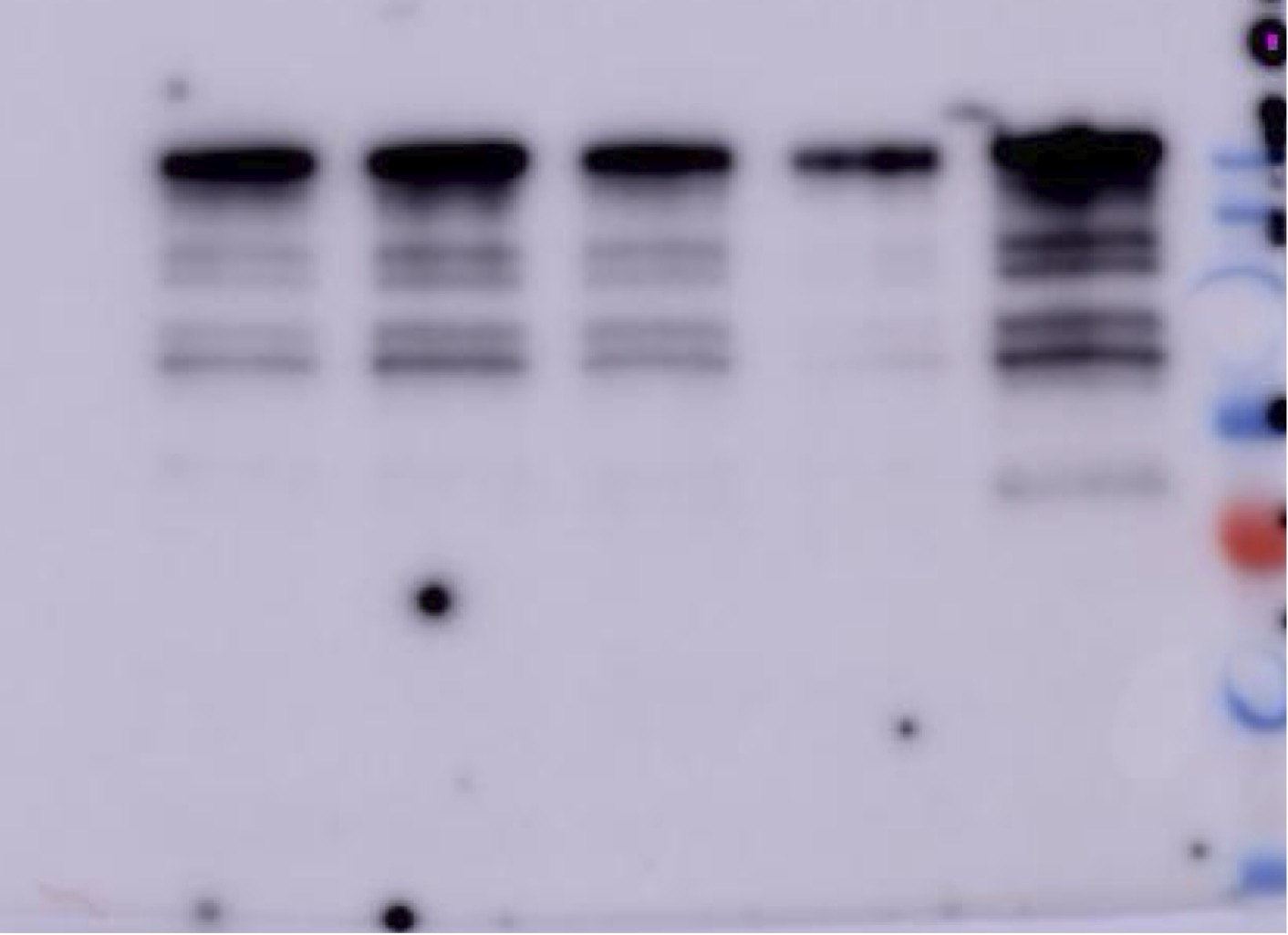

Supplement: Figure 5—figure supplement 2—source data 2. [file elife-110091-fig5-figsupp2-data2.zip › Figure 5 - Figure Supplement 2 - Source Data 2.tiff]

CTD  
Ser2P

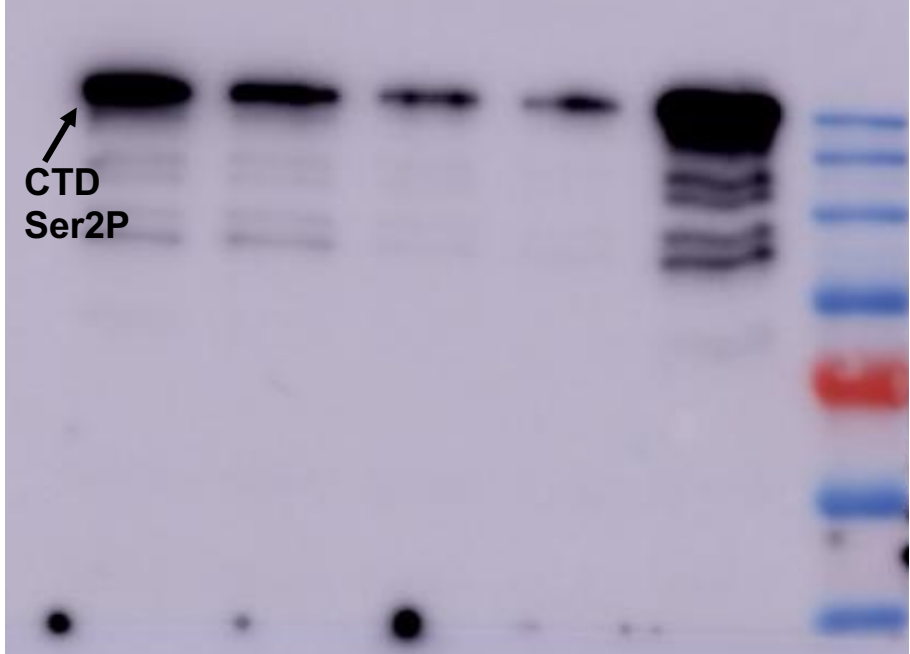

Supplement: Figure 5—figure supplement 2—source data 3. [file elife-110091-fig5-figsupp2-data3.zip › Figure 5 - Figure Supplement 2 - Source Data 3.pdf]

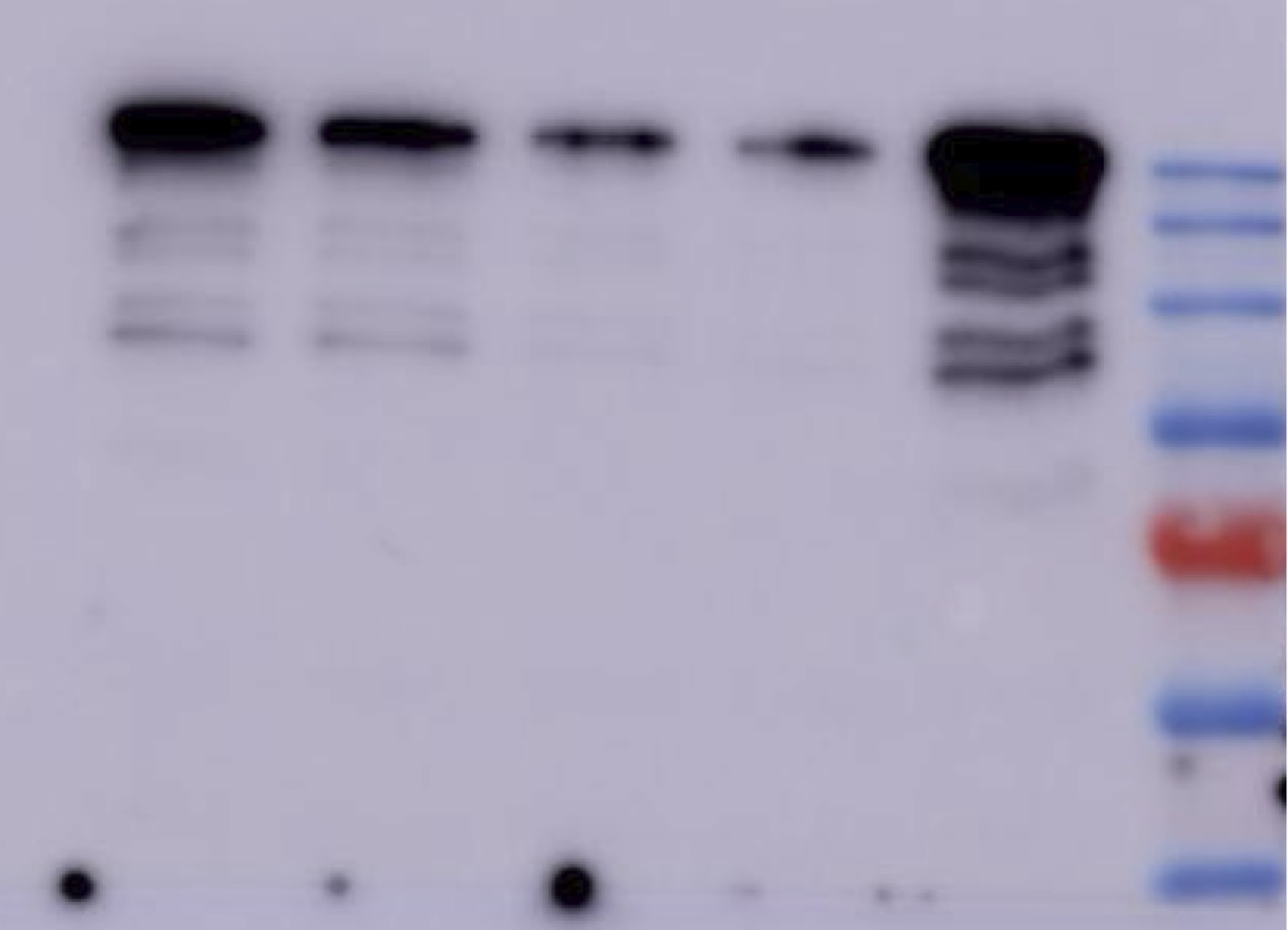

Supplement: Figure 5—figure supplement 2—source data 4. [file elife-110091-fig5-figsupp2-data4.zip › Figure 5 - Figure Supplement 2 - Source Data 4.tiff]

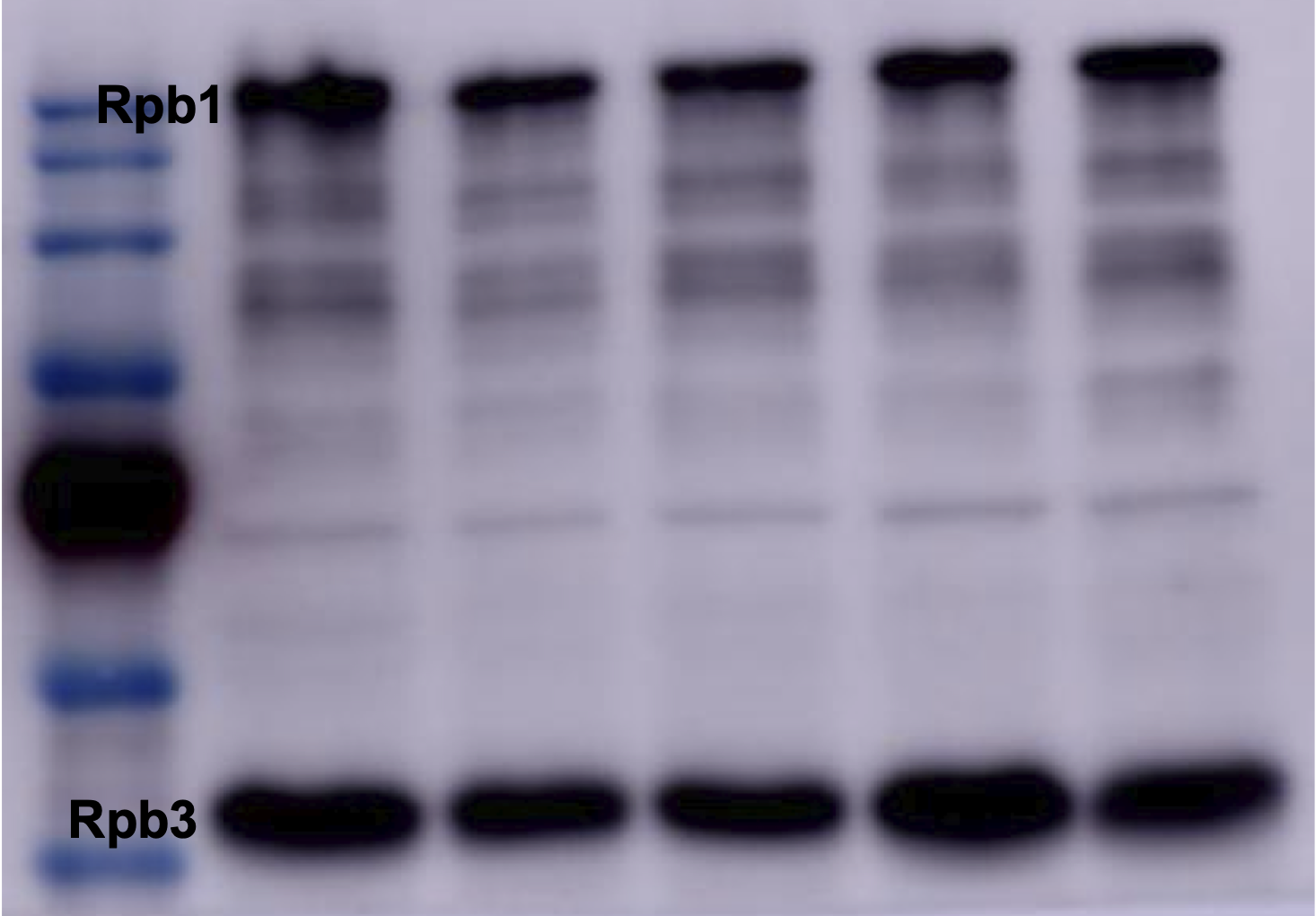

Supplement: Figure 5—figure supplement 2—source data 5. [file elife-110091-fig5-figsupp2-data5.zip › Figure 5 - Figure Supplement 2 - Source Data 5.tiff]

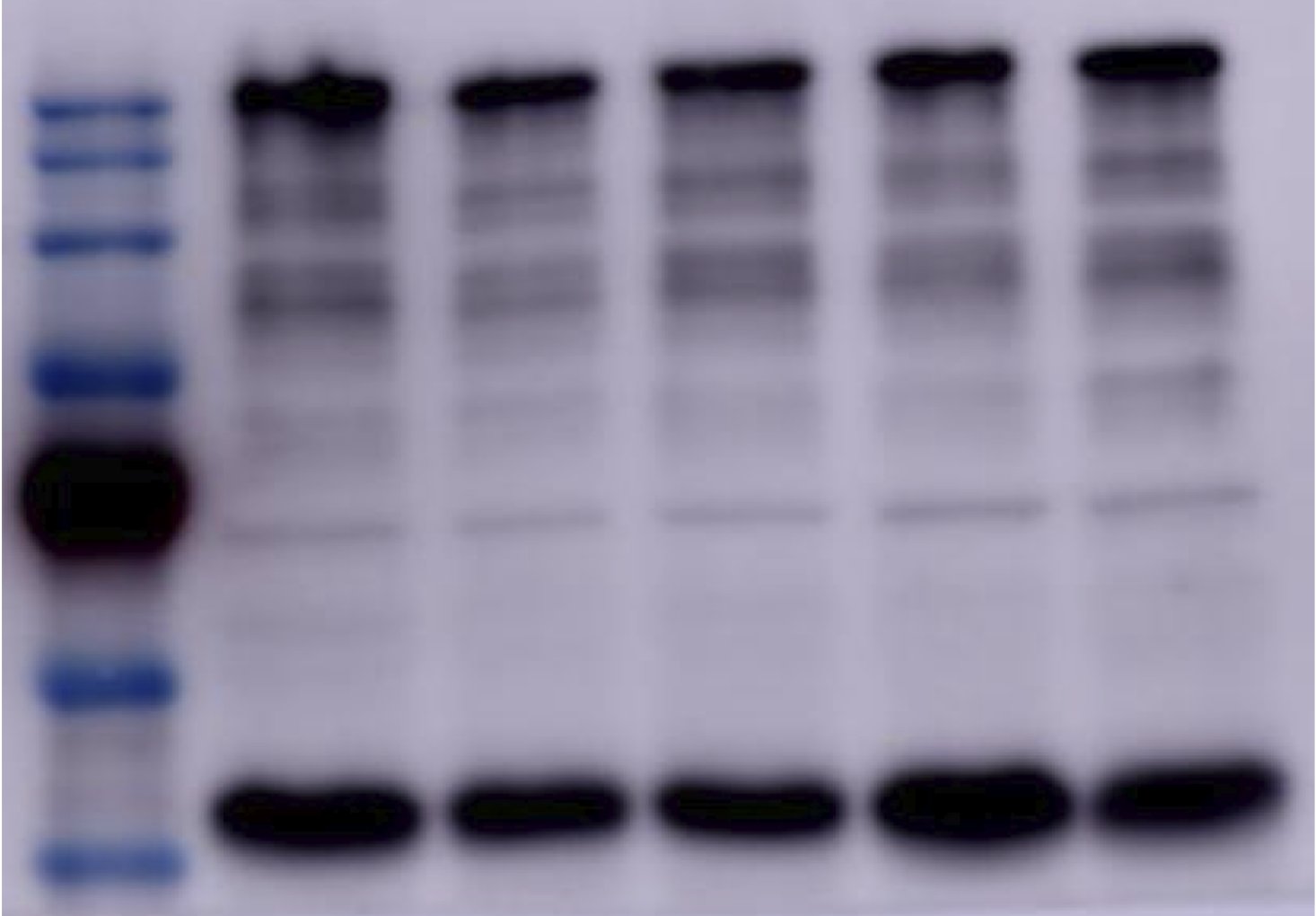

Supplement: Figure 5—figure supplement 2—source data 6. [file elife-110091-fig5-figsupp2-data6.zip › Figure 5 - Figure Supplement 2 - Source Data 6 .tiff]
