# Supplementary material for: Uncoupling the TFIIH Core and Kinase Modules leads to misregulated RNA polymerase II CTD Serine 5 phosphorylation
Source: eLife. 2026 Jun 8;15:RP110091. doi: 10.7554/eLife.110091 (PMC13246002; doi:10.7554/eLife.110091)
Supplement: Supplementary file 1. — Table A. Plasmids used in this study. Table B. Yeast strains used in this study. Table C. ChIP-seq parameters for S. cerevisiae reads. Table D. ChIP-seq parameters for S. pombe reads. [file elife-110091-supp1.pdf]

Supplementary File 1. Plasmids, yeast strains, and ChIP-seq parameters used in this study.
Table A. Plasmids used in this study.
Table B. Yeast strains used in this study.
Table C. ChIP-seq parameters for S. cerevisiae reads.
Table D. ChIP-seq parameters for S. pombe reads.

Table A. Plasmids used in this study.

Plasmid	Features	Construction or source reference
pSBEThis7-TFB3	T7 promoter driving N-terminal his-tagged TFB3 (1-285) [yeast TFIIH subunit], ArgU, KanR	962 bp NcoI-Bgl II fragment bearing TFB3 open reading frame from pBS-TFB3 ORF (SB835) was cloned into NcoI + BamHI sites of pSBEThis7. Created by Michael Keogh.
pBS-TFB3 A	TFB3 gene with 5' flanking sequence [yeast TFIIH subunit], f1+ ori, AmpR	TFB3 gene and promoter 2.059 kb fragment PCR'd from yeast genomic DNA using primers TFB3-A (O #411) and TFB3-C (O #413) and Pfu polymerase, and blunt ligated into SrfI site of pCR-Script SK+. Created by M. Keogh.
pRS415-KIN28	KIN28, LEU2, CEN/ARS, f1+ ori, AmpR	pRS426-KIN28 was digested with BamHI + HindIII. The ~1.3 kb fragment was cloned into BamHI + HindIII digested pRS415.
pBS-TFB3-BUR2	TFB3 promoter driving TFB3 (1-251)-BUR2 (2-395) fusion with TFB3 5' and 3' flanking sequence, f1+ ori, AmpR	~1.2 kb BUR2 fragment was amplified from pRS316-BUR2 (SB1209) using oligos Tfb3-Bur2 F (O #4287) and Bur2 stop Tfb3 3UTR R (O #4288). ~4.8 kb pBS-Tfb3 backbone was amplified using oligos Tfb3-3UTR F (O #4283) and Tfb3-1753 R (O #4284) on plasmid pBS-TFB3 A (SB836a). Fragments assembled through Gibson Assembly.
pBS-TFB3-CTK3	TFB3 promoter driving TFB3 (1-251)-CTK3 (2-296) fusion with TFB3 5' and 3' flanking sequence, f1+ ori, AmpR	~900 bp CTK3 fragment was amplified from pJYC4501 (F716) using oligos Tfb3-Ctk3 F (O #4285) and Ctk3 stop Tfb3 3UTR R (O #4286). ~4.8 kb pBS-Tfb3 backbone was amplified using oligos Tfb3-3UTR F (O #4283) and Tfb3-1753 R (O #4284) on plasmid pBS-TFB3 A (SB836a). Fragments assembled through Gibson Assembly.
pRS425-TFB3 (1-144)	TFB3 (1-144), LEU2, 2 μ ori, f1+ ori, AmpR	Oligos Tfb3- 3UTR F (O #4283) and TFB3 Ile144-Stop (O #4335) were used for inverse PCR on pRS425-TFB3 (SB1968). The resulting ~8.2 kb fragment was ligated by intramolecular Gibson isothermal assembly.
pRS425-TFB3 (1-251)	TFB3 (1-251), LEU2, 2 μ ori, f1+ ori, AmpR	Oligos Tfb3- 3UTR F (O #4283) and TFB3 Leu251-Stop (O #4336) were used for inverse PCR on pRS425-TFB3 (SB1968). The resulting ~8.5 kb fragment was ligated by intramolecular Gibson isothermal assembly.
pRS425-TFB3	TFB3, LEU2, 2 μ ori, f1+ ori, AmpR	~1.9 kb BamHI-Sal I fragment from pBS-TFB3 A (SB836a) cloned into the BamHI - Sal I sites of pRS425 (YV27).
pRS425-TFB3-MPK1	TFB3 promoter driving TFB3 (1-251)-MPK1 (2-484) fusion with TFB3 5' and 3' flanking sequence, LEU2, 2 μ ori, f1+ ori, AmpR	~8.5 kb pRS425-Tfb3 backbone fragment was amplified from pRS425-TFB3 (SB1968) using oligos Tfb3- 3UTR F (O #4283) and Tfb3- 1753 R (O #4284). ~1.5 kb Mpk1 fragment was amplified from genomic DNA with oligos Tfb3-Mpk1 for (O #4323) and Tfb3-Mpk1 rev (O #4324). Fragments were assembled through Gibson isothermal assembly.
pRS425-TFB3-BUR2	TFB3 promoter driving TFB3 (1-251)-BUR2 (2-395) fusion with TFB3 5' and 3' flanking sequence, LEU2, 2 μ ori, f1+ ori, AmpR	~3 kb fragment was amplified from pBS-TFB3-BUR2 (SB1973) and using oligos T3 sequencing primer (O #242) and KS poly: EcoRV-EcoRI-Pst (O #4260). ~6.8 kb pRS425 (YV27) backbone was digested with BamHI and SacI. Fragments assembled through Gibson isothermal assembly.
pRS425-TFB3-CTK3	TFB3 promoter driving TFB3 (1-251)-CTK3 (2-296) fusion with TFB3 5' and 3' flanking	~2.7 kb fragment was amplified from pBS-Tfb3-CTK3 (SB1974) using oligos T3 sequencing primer (O #242) and KS poly: EcoRV-EcoRI-Pst (O #4260). ~6.8 kb

	sequence, LEU2, 2 μ ori, f1+ ori, AmpR	pRS425 (YV27) backbone was digested with BamHI and SacI. Fragments assembled through Gibson isothermal assembly.
pRS424-TFB3	TFB3, TRP1, 2 μ ori, f1+ ori, AmpR	~1.9 kb BamHI-Sal I fragment from pRS425-TFB3 (SB1968) cloned into the BamHI - Sal I sites of pRS424 (YV26).
pRS424-TFB3 (1-251)	TFB3 (1-251), TRP1, 2 μ ori, f1+ ori, AmpR	~1.7 kb BamHI-SalI fragment from pRS425-TFB3 (1-251) (SB1994) and was cloned into the BamHI - Sal I sites of pRS424 (YV26).
pRS424-TFB3 (1-144)	TFB3 (1-144), TRP1, 2 μ ori, f1+ ori, AmpR	~1.4 kb BamHI-SalI fragment from pRS425-TFB3 (1-144) (SB1991) was cloned into the BamHI - Sal I sites of pRS424 (YV26).
pRS425	LEU2, 2 μ ori, f1+ ori, pBluescriptII SK polylinker with T7 and T3 promoters flanking, blue-white color selection, AmpR	Sikorski and Hieter (1989) Genetics 122: 19-27.
pRS315	LEU2, CEN/ARS, f1+ ori, pBluescript KS+ polylinker, blue-white color selection, AmpR	Sikorski and Hieter (1989) Genetics 122: 19-27.
pRS425-TFB3 (1-11, 238-Stop)	TFB3 (1-11, 238-Stop), LEU2, 2 μ ori, f1+ ori, AmpR	Oligos TFB3 33 R (O #4334) and TFB3 Asp11-Pro238 (O #4339) were used for inverse PCR on pRS425-TFB3 (SB1968). The resulting ~8 kb fragment was ligated by intramolecular Gibson isothermal assembly.
pRS315-TFB3 (1-11, 238-Stop)-Flag1-TAP	TFB3 (1-11, 238-Stop)-Flag1-TAP, LEU2, CEN/ARS, f1+ ori, AmpR	Primers TFB3 33 R (O #4334) and TFB3 Asp11-Pro238 (O #4339) were used for inverse PCR on pSH1542 (F1255). The resulting ~7.3 kb fragment was ligated by intramolecular Gibson assembly.
pRS425-TFB3 (1-11, 139-Stop)	TFB3 (1-11, 139-Stop), LEU2, 2 μ ori, f1+ ori, AmpR	Primers TFB3 33 R (O #4334) and TFB3 Asp11-Leu139 (O #4337) were used for inverse PCR on pRS425-TFB3 (SB1968). The resulting ~8.3 kb fragment was ligated by intramolecular Gibson assembly.
pLH366	TFB3 (Δ8-75)-Flag1-TAP, LEU2, CEN/ARS, AmpR	Warfield et al. (2016) MCB 36 (19): 2464-2475.
pSH1597	TFB3, URA3, CEN/ARS, AmpR	Warfield et al. (2016) MCB 36 (19): 2464-2475.
pSH1542	TFB3-Flag1-TAP, LEU2, CEN/ARS, AmpR	Warfield et al. (2016) MCB 36 (19): 2464-2475.
pRS315/TFB3Δ2	TFB3Δ2 (1-275), LEU2, CEN/ARS, AmpR, f1+ ori	Feaver et al. (2000) J. Biol. Chem. 275, 5941-5946

Table B. Yeast strains used in this study.

Strain	Genotype
**YSB744**	*MATα ura3-1 leu2-3,112 trp1-1 his3-11,15 kin28Δ::leu2Δ::TRP1 ade2-1 ade3-22 can1-100* [pRS426-KIN28]
**SHY907/ YF2456**	*MATα ura3Δ0 leu2Δ0 trp1Δ63 his3Δ200 ade2Δ::hisG lys2Δ0 met15Δ0 tfb3Δ::HygR tfb6Δ::KanMX* [pSH1597]. From Warfield et al. (2016) MCB 36 (19): 2464-2475.
**YSB3707**	*MATα ura3Δ0 leu2Δ0 trp1Δ63 his3Δ200 ade2Δ::hisG lys2Δ0 met15Δ0 tfb3Δ::HygR tfb6Δ::KanMX* [pSH1542]
**YSB3710**	*MATα ura3Δ0 leu2Δ0 trp1Δ63 his3Δ200 ade2Δ::hisG lys2Δ0 met15Δ0 tfb3Δ::HygR tfb6Δ::KanMX* [pRS425-TFB3 (1-144)]
**YSB3712**	*MATα ura3Δ0 leu2Δ0 trp1Δ63 his3Δ200 ade2Δ::hisG lys2Δ0 met15Δ0 tfb3Δ::HygR tfb6Δ::KanMX* [pRS425-TFB3]
**YSB3713**	*MATα ura3Δ0 leu2Δ0 trp1Δ63 his3Δ200 ade2Δ::hisG lys2Δ0 met15Δ0 tfb3Δ::HygR tfb6Δ::KanMX* [pRS425-TFB3-MPK1]
**YSB3715**	*MATα ura3Δ0 leu2Δ0 trp1Δ63 his3Δ200 ade2Δ::hisG lys2Δ0 met15Δ0 tfb3Δ::HygR tfb6Δ::KanMX* [pRS425-TFB3-BUR2]
**YSB3717**	*MATα ura3Δ0 leu2Δ0 trp1Δ63 his3Δ200 ade2Δ::hisG lys2Δ0 met15Δ0 tfb3Δ::HygR tfb6Δ::KanMX* [pRS425-TFB3-CTK3]
**YSB3722**	*MATα ura3Δ0 leu2Δ0 trp1Δ63 his3Δ200 ade2Δ::hisG lys2Δ0 met15Δ0 tfb3Δ::HygR tfb6Δ::KanMX* [pRS424-TFB3 (1-251)]
**YSB3704**	*MATα ura3Δ0 leu2Δ0 trp1Δ63 his3Δ200 ade2Δ::hisG lys2Δ0 met15Δ0 tfb3Δ::HygR tfb6Δ::KanMX* [pRS424-TFB3 (1-144)]
**YSB3723**	*MATα ura3Δ0 leu2Δ0 trp1Δ63 his3Δ200 ade2Δ::hisG lys2Δ0 met15Δ0 tfb3Δ::HygR tfb6Δ::KanMX* [pRS424-TFB3 (1-144), pSH1542]
**YSB3724**	*MATα ura3Δ0 leu2Δ0 trp1Δ63 his3Δ200 ade2Δ::hisG lys2Δ0 met15Δ0 tfb3Δ::HygR tfb6Δ::KanMX* [pRS424-TFB3 (1-144), pRS425]
**YSB3725**	*MATα ura3Δ0 leu2Δ0 trp1Δ63 his3Δ200 ade2Δ::hisG lys2Δ0 met15Δ0 tfb3Δ::HygR tfb6Δ::KanMX* [pRS424-TFB3 (1-144), pRS425-TFB3 (1-11, 238-Stop)]
**YSB3726**	*MATα ura3Δ0 leu2Δ0 trp1Δ63 his3Δ200 ade2Δ::hisG lys2Δ0 met15Δ0 tfb3Δ::HygR tfb6Δ::KanMX* [pRS424-TFB3 (1-144), pRS315-TFB3 (1-11, 238-Stop)-Flag1-TAP]
**YSB3727**	*MATα ura3Δ0 leu2Δ0 trp1Δ63 his3Δ200 ade2Δ::hisG lys2Δ0 met15Δ0 tfb3Δ::HygR tfb6Δ::KanMX* [pRS424-TFB3 (1-144), pRS425-TFB3 (1-11, 139-Stop)]
**YSB3728**	*MATα ura3Δ0 leu2Δ0 trp1Δ63 his3Δ200 ade2Δ::hisG lys2Δ0 met15Δ0 tfb3Δ::HygR tfb6Δ::KanMX* [pRS424-TFB3 (1-144), pLH366]
**YSB3729**	*MATα ura3Δ0 leu2Δ0 trp1Δ63 his3Δ200 ade2Δ::hisG lys2Δ0 met15Δ0 tfb3Δ::HygR tfb6Δ::KanMX* [pRS424-TFB3 (1-251), pSH1542]
**YSB3730**	*MATα ura3Δ0 leu2Δ0 trp1Δ63 his3Δ200 ade2Δ::hisG lys2Δ0 met15Δ0 tfb3Δ::HygR tfb6Δ::KanMX* [pRS424-TFB3 (1-251), pRS425]
**YSB3731**	*MATα ura3Δ0 leu2Δ0 trp1Δ63 his3Δ200 ade2Δ::hisG lys2Δ0 met15Δ0 tfb3Δ::HygR tfb6Δ::KanMX* [pRS424-TFB3 (1-251), pRS425-TFB3 (1-11, 238-Stop)]
**YSB3732**	*MATα ura3Δ0 leu2Δ0 trp1Δ63 his3Δ200 ade2Δ::hisG lys2Δ0 met15Δ0 tfb3Δ::HygR tfb6Δ::KanMX* [pRS424-TFB3 (1-251), pRS315-TFB3 (1-11, 238-Stop)-Flag1-TAP]
**YSB3733**	*MATα ura3Δ0 leu2Δ0 trp1Δ63 his3Δ200 ade2Δ::hisG lys2Δ0 met15Δ0 tfb3Δ::HygR tfb6Δ::KanMX* [pRS424-TFB3 (1-251), pRS425-TFB3 (1-11, 139-Stop)]
**YSB3734**	*MATα ura3Δ0 leu2Δ0 trp1Δ63 his3Δ200 ade2Δ::hisG lys2Δ0 met15Δ0 tfb3Δ::HygR tfb6Δ::KanMX* [pRS424-TFB3 (1-251), pLH366]
**YSB3786**	*MATα ura3-1 leu2-3,112 trp1-1 his3-11,15 kin28Δ::leu2Δ::TRP1 ade2-1 ade3-22 can1-100* [pRS415-KIN28]
**YSB3787**	*MATα ura3Δ0 leu2Δ0 trp1Δ63 his3Δ200 ade2Δ::hisG lys2Δ0 met15Δ0 tfb3Δ::HygR tfb6Δ::KanMX* [pRS424-TFB3]
**YSB3788**	*MATα ura3Δ0 leu2Δ0 trp1Δ63 his3Δ200 ade2Δ::hisG lys2Δ0 met15Δ0 tfb3Δ::HygR tfb6Δ::KanMX* [pRS424-TFB3, pRS425]
**YSB207**	*MATa ura3-52 leu2-3,112 his3Δ200 tfb1Δ::LEU2* [pRS316-TFB1] From Matsui et al., (1995) Nucleic Acids Res 23, 767-772.

**YSB260**	*MATa ura3-52 leu2-3,112 his3Δ200 tfb1Δ::LEU2* [pRS313-tfb1-101] From Matsui et al., (1995) Nucleic Acids Res 23, 767-772.

Table C: ChIP-seq parameters for S. cerevisiae reads

Sample	Total reads	Mapped reads	% Mapped reads	Deduplicated reads	% Deduplicated reads	Fragments average size	Fragments size std	Pearson correlation
CJ1_ChIPseq_Input_Tfb3WT_Spike_R1_20221206	28060948	24316268	86.66%	17666200	62.96%	187.151693	54.84188419	
CJ2_ChIPseq_Input_Tfb3WT_Spike_R2_20221206	25142766	21683658	86.24%	16285104	64.77%	183.7562841	52.72211465	
CJ3_ChIPseq_Input_tfb3N-LC_Spike_R1_20221206	30338000	28318312	93.34%	19901330	65.60%	182.0606635	51.85993552	
CJ4_ChIPseq_Input_tfb3N-LC_Spike_R2_20221206	25623514	23744682	92.67%	18112754	70.69%	193.12372	59.31659928	
CJ5_ChIPseq_Input_tfb3N-C_Spike_R1_20221206	26168988	23924892	91.42%	18380502	70.24%	185.0257088	53.5344394	
CJ6_ChIPseq_Input_tfb3N-C_Spike_R2_20221206	28575464	26292900	92.01%	20354770	71.23%	192.773337	58.00570866	
CJ7_ChIPseq_Input_tfb3NL-C_Spike_R1_20221206	31451666	27197436	86.47%	19478246	61.93%	185.1408434	54.21921369	
CJ8_ChIPseq_Input_tfb3NL-C_Spike_R2_20221206	27660176	24037478	86.90%	17810316	64.39%	187.0205919	55.0058291	
CJ9_ChIPseq_IP_Tfb1_Tfb3WT_Spike_R1_20221206	21933424	18706082	85.29%	8523750	38.86%	178.9884375	50.4586975	
CJ11_ChIPseq_IP_Tfb1_Tfb3WT_Spike_R2_20221206	31388434	27114844	86.38%	15064408	47.99%	180.6129835	50.89617922	
CJ10_ChIPseq_IP_Kin28_Tfb3WT_Spike_R1_20221206	25212688	22084698	87.59%	11771758	46.69%	183.0545385	53.52558161	
CJ12_ChIPseq_IP_Kin28_Tfb3WT_Spike_R2_20221206	27504962	24137616	87.76%	12353214	44.91%	187.8508748	55.94383818	
CJ13_ChIPseq_IP_Tfb1_tfb3N-LC_Spike_R1_20221206	26079084	24104222	92.43%	14991702	57.49%	186.3978388	54.85202601	
CJ15_ChIPseq_IP_Tfb1_tfb3N-LC_Spike_R2_20221206	28492222	26201452	91.96%	16217586	56.92%	184.8119941	53.90919534	
CJ14_ChIPseq_IP_Kin28_tfb3N-LC_Spike_R1_20221206	26720222	24929216	93.30%	15482036	57.94%	186.9584852	55.33301124	
CJ16_ChIPseq_IP_Kin28_tfb3N-LC_Spike_R2_20221206	30556826	28384914	92.89%	16973328	55.55%	191.2947476	56.93516834	
CJ17_ChIPseq_IP_Tfb1_tfb3N-C_Spike_R1_20221206	28014174	25070362	89.49%	12797216	45.68%	186.2883061	55.37930129	
CJ19_ChIPseq_IP_Tfb1_tfb3N-C_Spike_R2_20221206	23685496	20727300	87.51%	7118384	30.05%	181.9331486	52.28734443	
CJ18_ChIPseq_IP_Kin28_tfb3N-C_Spike_R1_20221206	26253552	23887458	90.99%	13124260	49.99%	190.0250035	55.02351744	
CJ20_ChIPseq_IP_Kin28_tfb3N-C_Spike_R2_20221206	27987324	25315522	90.45%	10788264	38.55%	186.4347784	54.96583003	
CJ21_ChIPseq_IP_Tfb1_tfb3NL-C_Spike_R1_20221206	21097654	17251128	81.77%	5973408	28.31%	184.6293489	52.40626339	
CJ23_ChIPseq_IP_Tfb1_tfb3NL-C_Spike_R2_20221206	25005018	21356392	85.41%	7346354	29.38%	182.4302594	52.97200481	
CJ22_ChIPseq_IP_Kin28_tfb3NL-C_Spike_R1_20221206	22756574	19667140	86.42%	10115178	44.45%	181.6662182	52.00454101	
CJ24_ChIPseq_IP_Kin28_tfb3NL-C_Spike_R2_20221206	24373472	21125784	86.68%	8198594	33.64%	197.0143442	60.81813163	
CJ25_ChIPseq_IP_8WG16_Tfb3WT_Spike_R1_20221206	27441896	23822720	86.81%	18410874	67.09%	193.5061943	56.02529553	
CJ28_ChIPseq_IP_8WG16_Tfb3WT_Spike_R2_20221206	25996340	22584706	86.88%	17075896	65.69%	194.006966	55.81883403	
CJ26_ChIPseq_IP_3E8_Tfb3WT_Spike_R1_20221206	25838296	21654764	83.81%	14876982	57.58%	184.3699006	52.06012194	
CJ29_ChIPseq_IP_3E8_Tfb3WT_Spike_R2_20221206	28040716	21924548	78.19%	15023372	53.58%	183.4677026	52.19602674	
CJ31_ChIPseq_IP_8WG16_tfb3N-LC_Spike_R1_20221206	30495908	27080862	88.80%	18984972	62.25%	189.7476167	55.15932956	
CJ34_ChIPseq_IP_8WG16_tfb3N-LC_Spike_R2_20221206	25196604	22133444	87.84%	16020930	63.58%	189.3908446	54.63351052	
CJ32_ChIPseq_IP_3E8_tfb3N-LC_Spike_R1_20221206	27969570	25873014	92.50%	16869978	60.32%	191.6410608	55.85065663	
CJ35_ChIPseq_IP_3E8_tfb3N-LC_Spike_R2_20221206	22792800	20375434	89.39%	13537306	59.39%	186.0942068	53.96482432	
CJ37_ChIPseq_IP_8WG16_tfb3N-C_Spike_R1_20221206	27586338	24259098	87.94%	17339088	62.85%	185.0421983	51.76648932	
CJ40_ChIPseq_IP_8WG16_tfb3N-C_Spike_R2_20221206	23630278	20684686	87.53%	14216836	60.16%	192.4918004	53.96706389	
CJ38_ChIPseq_IP_3E8_tfb3N-C_Spike_R1_20221206	25817692	22883660	88.64%	14284602	55.33%	181.8681642	51.2903419	

CJ41_ChIPseq_IP_3E8_tfb3N-C_Spike_R2_20221206	22984208	20364348	88.60%	10643430	46.31%	179.9475536	48.44600528	
CJ43_ChIPseq_IP_8WG16_tfb3NL-C_Spike_R1_20221206	31686578	26214330	82.73%	18765146	59.22%	198.0127415	55.85851478	
CJ46_ChIPseq_IP_8WG16_tfb3NL-C_Spike_R2_20221206	28413026	23563850	82.93%	16894050	59.46%	199.4182276	56.07545824	
CJ44_ChIPseq_IP_3E8_tfb3NL-C_Spike_R1_20221206	22729102	18672918	82.15%	10778412	47.42%	194.0807191	56.0081025	
CJ47_ChIPseq_IP_3E8_tfb3NL-C_Spike_R2_20221206	27089408	22290936	82.29%	12664346	46.75%	190.8298633	54.2312006	
ChIPseq_Input_Tfb3WT_Spike_CJ1-CJ2_20221206				33951304		185.523049	53.86224722	0.9987
ChIPseq_Input_tfb3N-LC_Spike_CJ3-CJ4_20221206				38014084		187.3319314	55.81203566	0.9929
ChIPseq_Input_tfb3N-C_Spike_CJ5-CJ6_20221206				38735272		189.0969643	56.06225203	0.9982
ChIPseq_Input_tfb3NL-C_Spike_CJ7-CJ8_20221206				37288562		186.0386767	54.60441417	0.9918
ChIPseq_IP_Tfb1_Tfb3WT_Spike_CJ9-CJ11_20221206				23588158		180.0259422	50.74452676	0.9937
ChIPseq_IP_Kin28_Tfb3WT_Spike_CJ10-CJ12_20221206				24124972		185.5105069	54.82962941	0.9994
ChIPseq_IP_Tfb1_tfb3N-LC_Spike_CJ13-CJ15_20221206				31209288		185.5737709	54.36990612	0.9955
ChIPseq_IP_Kin28_tfb3N-LC_Spike_CJ14-CJ16_20221206				32455364		189.2262399	56.21833234	0.9948
ChIPseq_IP_Tfb1_tfb3N-C_Spike_CJ17-CJ19_20221206				19915600		184.7316529	54.334475	0.988
ChIPseq_IP_Kin28_tfb3N-C_Spike_CJ18-CJ20_20221206				23912524		188.4052541	55.02650556	0.9945
ChIPseq_IP_Tfb1_tfb3NL-C_Spike_CJ21-CJ23_20221206				13319762		183.4164677	52.73038151	0.9976
ChIPseq_IP_Kin28_tfb3NL-C_Spike_CJ22-CJ24_20221206				18313772		188.5371705	56.63808578	0.9967
ChIPseq_IP_8WG16_Tfb3WT_Spike_CJ25-CJ28_20221206				35486770		193.7471609	55.92660148	0.9982
ChIPseq_IP_3E8_Tfb3WT_Spike_CJ26-CJ29_20221206				29900354		183.916593	52.13040131	0.9802
ChIPseq_IP_8WG16_tfb3N-LC_Spike_CJ31-CJ34_20221206				35005902		189.5843351	54.91959214	0.9305
ChIPseq_IP_3E8_tfb3N-LC_Spike_CJ32-CJ35_20221206				30407284		189.1716045	55.08808583	0.9763
ChIPseq_IP_8WG16_tfb3N-C_Spike_CJ37-CJ40_20221206				31555924		188.3984547	52.89928193	0.9869
ChIPseq_IP_3E8_tfb3N-C_Spike_CJ38-CJ41_20221206				24928032		181.0481281	50.10467126	0.9863
ChIPseq_IP_8WG16_tfb3NL-C_Spike_CJ43-CJ46_20221206				35659196		198.6786105	55.96579816	0.985
ChIPseq_IP_3E8_tfb3NL-C_Spike_CJ44-CJ47_20221206				23442758		192.324528	55.07913115	0.9835

Table D: ChIP-seq parameters for S. pombe reads

Sample	Total reads	Mapped reads	% Mapped reads	Deduplicated reads	% Deduplicated reads	Fragments average size	Fragments size std
CJ1_ChIPseq_Input_Tfb3WT_Spike_R1_20221206	28060948	846652	3.02%	623366	2.22%	185.1013305	53.83830897
CJ2_ChIPseq_Input_Tfb3WT_Spike_R2_20221206	25142766	781614	3.11%	596604	2.37%	181.5391013	51.55880425
CJ3_ChIPseq_Input_tfb3N-LC_Spike_R1_20221206	30338000	695654	2.29%	492432	1.62%	179.9999756	50.50852705
CJ4_ChIPseq_Input_tfb3N-LC_Spike_R2_20221206	25623514	560076	2.19%	428710	1.67%	191.1615171	58.56076855
CJ5_ChIPseq_Input_tfb3N-C_Spike_R1_20221206	26168988	670838	2.56%	519310	1.98%	182.5892319	52.50833339
CJ6_ChIPseq_Input_tfb3N-C_Spike_R2_20221206	28575464	645568	2.26%	503894	1.76%	189.4855466	56.74773664
CJ7_ChIPseq_Input_tfb3NL-C_Spike_R1_20221206	31451666	1368696	4.35%	990526	3.15%	183.7836241	53.83598574
CJ8_ChIPseq_Input_tfb3NL-C_Spike_R2_20221206	27660176	1332618	4.82%	995828	3.60%	185.958535	54.71051443
CJ9_ChIPseq_IP_Tfb1_Tfb3WT_Spike_R1_20221206	21933424	732152	3.34%	335374	1.53%	176.9659902	49.48895332
CJ11_ChIPseq_IP_Tfb1_Tfb3WT_Spike_R2_20221206	31388434	1070344	3.41%	619132	1.97%	177.9171195	49.28553322
CJ10_ChIPseq_IP_Kin28_Tfb3WT_Spike_R1_20221206	25212688	674476	2.68%	379710	1.51%	179.4113244	51.7300538
CJ12_ChIPseq_IP_Kin28_Tfb3WT_Spike_R2_20221206	27504962	747658	2.72%	408752	1.49%	184.1809263	54.14960845
CJ13_ChIPseq_IP_Tfb1_tfb3N-LC_Spike_R1_20221206	26079084	728480	2.79%	457532	1.75%	183.5296854	53.51132098
CJ15_ChIPseq_IP_Tfb1_tfb3N-LC_Spike_R2_20221206	28492222	790138	2.77%	494990	1.74%	181.7431908	52.49740535
CJ14_ChIPseq_IP_Kin28_tfb3N-LC_Spike_R1_20221206	26720222	666442	2.49%	422284	1.58%	183.0614989	53.28236879
CJ16_ChIPseq_IP_Kin28_tfb3N-LC_Spike_R2_20221206	30556826	719998	2.36%	434818	1.42%	187.2660699	55.15772767
CJ17_ChIPseq_IP_Tfb1_tfb3N-C_Spike_R1_20221206	28014174	1063794	3.80%	544726	1.94%	182.2505076	53.30852239
CJ19_ChIPseq_IP_Tfb1_tfb3N-C_Spike_R2_20221206	23685496	1072190	4.53%	362356	1.53%	178.0362296	50.24777303
CJ18_ChIPseq_IP_Kin28_tfb3N-C_Spike_R1_20221206	26253552	767778	2.92%	433724	1.65%	185.1550525	53.05492754
CJ20_ChIPseq_IP_Kin28_tfb3N-C_Spike_R2_20221206	27987324	834902	2.98%	372238	1.33%	180.7236016	51.9326008
CJ21_ChIPseq_IP_Tfb1_tfb3NL-C_Spike_R1_20221206	21097654	1141534	5.41%	378344	1.79%	182.2998065	51.77239721
CJ23_ChIPseq_IP_Tfb1_tfb3NL-C_Spike_R2_20221206	25005018	1487086	5.95%	495676	1.98%	180.1907738	52.38100476
CJ22_ChIPseq_IP_Kin28_tfb3NL-C_Spike_R1_20221206	22756574	1136130	4.99%	610830	2.68%	177.3449569	49.83973602
CJ24_ChIPseq_IP_Kin28_tfb3NL-C_Spike_R2_20221206	24373472	1178548	4.84%	458122	1.88%	192.6899821	58.951525
CJ25_ChIPseq_IP_8WG16_Tfb3WT_Spike_R1_20221206	27441896	1128564	4.11%	884000	3.22%	190.3345271	54.68234901
CJ28_ChIPseq_IP_8WG16_Tfb3WT_Spike_R2_20221206	25996340	1092832	4.20%	835682	3.21%	190.8983728	54.47781093
CJ26_ChIPseq_IP_3E8_Tfb3WT_Spike_R1_20221206	25838296	1662118	6.43%	1154128	4.47%	181.3311574	50.62594455
CJ29_ChIPseq_IP_3E8_Tfb3WT_Spike_R2_20221206	28040716	1958358	6.98%	1357596	4.84%	180.5729215	50.74377243
CJ27_ChIPseq_IP_3E10_Tfb3WT_Spike_R1_20221206	30278142	3027838	10.00%	507444	1.68%	180.0213304	51.4626712
CJ30_ChIPseq_IP_3E10_Tfb3WT_Spike_R2_20221206	31671338	3343260	10.56%	484610	1.53%	178.9929964	52.28392339
CJ31_ChIPseq_IP_8WG16_tfb3N-LC_Spike_R1_20221206	30495908	2113824	6.93%	1484396	4.87%	185.3269855	53.05985514
CJ34_ChIPseq_IP_8WG16_tfb3N-LC_Spike_R2_20221206	25196604	1773960	7.04%	1293002	5.13%	185.4773775	52.69852257
CJ32_ChIPseq_IP_3E8_tfb3N-LC_Spike_R1_20221206	27969570	1003486	3.59%	657046	2.35%	187.0917653	53.92475137
CJ35_ChIPseq_IP_3E8_tfb3N-LC_Spike_R2_20221206	22792800	1243894	5.46%	824336	3.62%	181.9736928	51.7804567

CJ33_ChIPseq_IP_3E10_tfb3N-LC_Spike_R1_20221206	42940988	1247534	2.91%	627904	1.46%	183.4230264	52.97077163
CJ36_ChIPseq_IP_3E10_tfb3N-LC_Spike_R2_20221206	36916590	1791252	4.85%	508930	1.38%	186.0975183	56.74859061
CJ37_ChIPseq_IP_8WG16_tfb3N-C_Spike_R1_20221206	27586338	1659278	6.01%	1199388	4.35%	180.2060768	49.49942587
CJ40_ChIPseq_IP_8WG16_tfb3N-C_Spike_R2_20221206	23630278	1365400	5.78%	950242	4.02%	187.1708954	51.92034006
CJ38_ChIPseq_IP_3E8_tfb3N-C_Spike_R1_20221206	25817692	1314274	5.09%	820672	3.18%	177.7428912	49.17595847
CJ41_ChIPseq_IP_3E8_tfb3N-C_Spike_R2_20221206	22984208	1090638	4.75%	568434	2.47%	175.8922302	46.61382335
CJ39_ChIPseq_IP_3E10_tfb3N-C_Spike_R1_20221206	51180370	2894724	5.66%	254696	0.50%	179.5519757	51.41823937
CJ42_ChIPseq_IP_3E10_tfb3N-C_Spike_R2_20221206	34504488	1583728	4.59%	258710	0.75%	183.0624792	52.30351672
CJ43_ChIPseq_IP_8WG16_tfb3NL-C_Spike_R1_20221206	31686578	2766612	8.73%	2016986	6.37%	194.0235222	54.1382237
CJ46_ChIPseq_IP_8WG16_tfb3NL-C_Spike_R2_20221206	28413026	2677376	9.42%	1948088	6.86%	195.6566202	54.61740573
CJ44_ChIPseq_IP_3E8_tfb3NL-C_Spike_R1_20221206	22729102	1896804	8.35%	1092670	4.81%	190.4241445	54.41026838
CJ47_ChIPseq_IP_3E8_tfb3NL-C_Spike_R2_20221206	27089408	2631848	9.72%	1495020	5.52%	187.6967934	52.80320334
CJ45_ChIPseq_IP_3E10_tfb3NL-C_Spike_R1_20221206	30375814	2941948	9.69%	455950	1.50%	181.3833798	54.21909823
CJ48_ChIPseq_IP_3E10_tfb3NL-C_Spike_R2_20221206	31921716	4194896	13.14%	340390	1.07%	181.5553042	51.34714194
ChIPseq_Input_Tfb3WT_Spike_CJ1-CJ2_20221206				1219970		183.3592875	52.76588264
ChIPseq_Input_tfb3N-LC_Spike_CJ3-CJ4_20221206				921142		185.1946844	54.68865897
ChIPseq_Input_tfb3N-C_Spike_CJ5-CJ6_20221206				1023204		185.9854379	54.74584347
ChIPseq_Input_tfb3NL-C_Spike_CJ7-CJ8_20221206				1986354		184.8739822	54.28704405
ChIPseq_IP_Tfb1_Tfb3WT_Spike_CJ9-CJ11_20221206				954506		177.5829319	49.3591389
ChIPseq_IP_Kin28_Tfb3WT_Spike_CJ10-CJ12_20221206				788462		181.8839665	53.0516712
ChIPseq_IP_Tfb1_tfb3N-LC_Spike_CJ13-CJ15_20221206				952522		182.601311	52.99430978
ChIPseq_IP_Kin28_tfb3N-LC_Spike_CJ14-CJ16_20221206				857102		185.1945276	54.282517
ChIPseq_IP_Tfb1_tfb3N-C_Spike_CJ17-CJ19_20221206				907082		180.5670116	52.14821133
ChIPseq_IP_Kin28_tfb3N-C_Spike_CJ18-CJ20_20221206				805962		183.1083624	52.58591834
ChIPseq_IP_Tfb1_tfb3NL-C_Spike_CJ21-CJ23_20221206				874020		181.1037276	52.12883967
ChIPseq_IP_Kin28_tfb3NL-C_Spike_CJ22-CJ24_20221206				1068952		183.9213922	54.46554015
ChIPseq_IP_8WG16_Tfb3WT_Spike_CJ25-CJ28_20221206				1719682		190.6085288	54.58374492
ChIPseq_IP_3E8_Tfb3WT_Spike_CJ26-CJ29_20221206				2511724		180.9213281	50.69105321
ChIPseq_IP_3E10_Tfb3WT_Spike_CJ27-CJ30_20221206				992054		179.518998	51.86796548
ChIPseq_IP_8WG16_tfb3N-LC_Spike_CJ31-CJ34_20221206				2777398		185.3969996	52.89198006
ChIPseq_IP_3E8_tfb3N-LC_Spike_CJ32-CJ35_20221206				1481382		184.2437413	52.80350633
ChIPseq_IP_3E10_tfb3N-LC_Spike_CJ33-CJ36_20221206				1136834		184.6203245	54.7103866
ChIPseq_IP_8WG16_tfb3N-C_Spike_CJ37-CJ40_20221206				2149630		183.2848686	50.70197932
ChIPseq_IP_3E8_tfb3N-C_Spike_CJ38-CJ41_20221206				1389106		176.985585	48.15256033
ChIPseq_IP_3E10_tfb3N-C_Spike_CJ39-CJ42_20221206				513406		181.3209507	51.89581686
ChIPseq_IP_8WG16_tfb3NL-C_Spike_CJ43-CJ46_20221206				3965074		194.8258827	54.38029447
ChIPseq_IP_3E8_tfb3NL-C_Spike_CJ44-CJ47_20221206				2587690		188.8484363	53.50462746
ChIPseq_IP_3E10_tfb3NL-C_Spike_CJ45-CJ48_20221206				796340		181.4568677	53.01054631
