# Supplementary material for: Uncoupling the TFIIH Core and Kinase Modules leads to misregulated RNA polymerase II CTD Serine 5 phosphorylation
Source: eLife. 2026 Jun 8;15:RP110091. doi: 10.7554/eLife.110091 (PMC13246002; doi:10.7554/eLife.110091)
Supplement: Peer review history

# Editor report: eLife Assessment

**DOI:** 10.7554/eLife.110091.4.sa0

**Authors:** Alan G Hinnebusch

**Affiliations:** Eunice Kennedy Shriver National Institute of Child Health and Human Development United States

**Keywords:** Important, Solid

This **important** work demonstrates the role of physically linking the core and CTD kinase modules of TFIIH via separate domains of subunit Tfb3 in confining RNA Polymerase II Serine 5 CTD phosphorylation to promoter regions of transcribed genes in budding yeast. The main findings, resulting from analyses of viable Tfb3 mutants in which the linkage between TFIIH core and kinase modules has been severed, are supported by **solid** evidence from in vitro and in vivo experiments. The new findings raise the intriguing possibility that the Tfb3-mediated connection between core and kinase modules of TFIIH is an evolutionary addition to an ancestral state of physically unconnected enzymes.

# Referee report: Reviewer #1 (Public review)

**DOI:** 10.7554/eLife.110091.4.sa1

[Editors' note: this version has been assessed by the Reviewing Editor without further input from the original reviewers. The authors have addressed the comments raised in the previous rounds of review.]

Giordano et al. demonstrate that yeast cells expressing separated N- and C-terminal regions of Tfb3 are viable and grow well. Using this creative and powerful tool, the authors effectively uncouple CTD Ser5 phosphorylation at promoters and assess its impact on transcription. This strategy is complementary to previous approaches, such as Kin28 depletion or the use of CDK7 inhibitors. The results are largely consistent with earlier studies, reinforcing the importance of the Tfb3 linkage in mediating CTD Ser5 phosphorylation at promoters and subsequent transcription.

Notably, the authors also observe effects attributable to the Tfb3 linker itself, beyond its role as a simple physical connection between the N- and C-terminal domains. These findings provide functional insight into the Tfb3 linker, which had previously been observed in structural studies but lacked clear functional relevance. Overall, I am very positive about the publication of this manuscript.

# Referee report: Reviewer #2 (Public review)

**DOI:** 10.7554/eLife.110091.4.sa2

Summary:

This work advances our understanding of how TFIIH coordinates DNA melting and CTD phosphorylation during transcription initiation. The finding that untethered kinase activity becomes "unfocused," phosphorylating the CTD at ser5 throughout the coding sequence rather than being promoter-restricted, suggests that the TFIIH Core-Kinase linkage not only targets the kinase to promoters but also constrains its activity in a spatial and temporal manner.

Strengths:

The experiments presented are straightforward and the model for coupling initiation and CTD phosphorylation and for evolution of these linked processes are interesting and novel. The results have important implications for the regulation of initiation and CTD phosphorylation.

# Referee report: Reviewer #3 (Public review)

**DOI:** 10.7554/eLife.110091.4.sa3

Summary:

Eukaryotic gene transcription requires a large assemblage of protein complexes that govern the molecular events required for RNA Polymerase II to produce mRNAs. One of these complexes, TFIIH, comprises two modules, one of which promotes DNA unwinding at promoters, while the other contains a kinase (Kin28 in yeast) that phosphorylates the repeated motif at the C-terminal domain (CTD) of the largest subunit of Pol II. Kin28 phosphorylation of Ser5 in the YSPTSPS motif of the CTD is normally highly localized at promoter regions, and marks the beginning of a cycle of phosphorylation events and accompanying protein association with the CTD during the transition from initiation to elongation.

The two modules of TFIIH are linked by Tfb3. Tfb3 consists of two globular regions, an N-terminal domain that contacts the Core module of TFIIH and a C-terminal domain that contacts the kinase module, connected by a linker. In this paper, Giordano et al. test the role of Tfb3 as a connector between the two modules of TFIIH in yeast. They show that while no or very slow growth occurs if only the C-terminal or N-terminal region of Tfb3 is present, near normal growth is observed when the two unlinked regions are expressed. Consistent with this result, the separate domains are shown to interact with the two distinct TFIIH modules. ChIP experiments show that the Core module of TFIIH maintains its localization at gene promoters when the Tfb3 domains are separated, while localization of the kinase module, and of Ser5 phosphorylation on the CTD of Pol II, is disrupted. Finally, the authors examine the effect of separating the Tfb3 domains on another function of TFIIH, namely nucleotide excision repair, and find little or no effect when only the N-terminal region of Tfb3 or the two unlinked domains are present.

Strengths:

Experiments involving expression of Tfb3 domains in yeast are well-controlled and the data regarding viability, interaction of the separate Tfb3 domains with TFIIH modules, genome-wide localization of the TFIIH modules and of phosphorylated Ser5 CTDs, and of effects on NER, are convincing. The experiments are consistent with current models of TFIIH structure and function and support a model in which Tfb3 tethers the kinase module of TFIIH close to initiation sites to prevent its promiscuous action on elongating Pol II.

# Author comment: Author response

**DOI:** 10.7554/eLife.110091.4.sa4

**Authors:** Gabriela Giordano, Robin Buratowski, Celia Jeronimo, Christian Poitras, François Robert, Stephen Buratowski

**Affiliations:** Harvard Medical School Boston United States; Harvard Medical School Boston United States; Institut de recherches cliniques de Montréal (IRCM) Montreal Canada; Institut de recherches cliniques de Montréal (IRCM) Montreal Canada; Institut de recherches cliniques de Montréal (IRCM) Montreal Canada; Harvard Medical School Boston United States

The following is the authors’ response to the previous reviews

**eLife Assessment**

This important work demonstrates the role of physically linking the core and CTD kinase modules of TFIIH via separate domains of subunit Tfb3 in confining RNA Polymerase II Serine 5 CTD phosphorylation to promoter regions of transcribed genes in budding yeast. The main findings, resulting from analyses of viable Tfb3 mutants in which the linkage between TFIIH core and kinase modules has been severed, are supported by solid evidence from in vitro and in vivo experiments. The new findings raise the intriguing possibility that the Tfb3-mediated connection between core and kinase modules of TFIIH is an evolutionary addition to an ancestral state of physically unconnected enzymes.

After consultation with the referees, we would like to suggest that you insert text into the RESULTS section acknowledging two limitations of your findings remaining in the revised manuscript, as follows:

(i) It remains possible that Kin28 abundance was reduced by splitting Tfb3, which could be a factor in reducing its occupancies at gene promoters.

In response, the paper now contains the following sentence:

“Kin28 levels in extracts were below the limit of detection for our antibody, so we cannot rule out that the drop in ChIP signal is partly due to reduced Kin28 levels in the split Tfb3 strains. However, the viability of the cells (Figure 2) and the Tfb3-TAP purifications (Figure 3) argue against a complete loss of Kin28.”

(ii) Lower than wild-type expression of the Tfb3 truncations might contribute to their mutant phenotypes shown in Figs. 2 & 5.

In response, the paper now contains the following sentence:

“There was some variation in protein expression levels (Figure 3A, left panel, lanes 1-4), and reduced levels of the split Tfb3 may contribute to the slow growth phenotypes.”

**Public Reviews:**

**Reviewer #1 (Public review):**

Giordano et al. demonstrate that yeast cells expressing separated N- and C-terminal regions of Tfb3 are viable and grow well. Using this creative and powerful tool, the authors effectively uncouple CTD Ser5 phosphorylation at promoters and assess its impact on transcription. This strategy is complementary to previous approaches, such as Kin28 depletion or the use of CDK7 inhibitors. The results are largely consistent with earlier studies, reinforcing the importance of the Tfb3 linkage in mediating CTD Ser5 phosphorylation at promoters and subsequent transcription.

Notably, the authors also observe effects attributable to the Tfb3 linker itself, beyond its role as a simple physical connection between the N- and C-terminal domains. These findings provide functional insight into the Tfb3 linker, which had previously been observed in structural studies but lacked clear functional relevance. Overall, I am very positive about the publication of this manuscript and offer a few minor comments below that may help to further strengthen the study.

We appreciate the reviewer’s positive assessment of our work and suggestions for improvement.

Page 4 PIC structures show the linker emerging from the N-terminal domain as a long alpha-helix running along the interface between the two ATPase subunits, followed by a turn and a short stretch of helix just N-terminal to a disordered region that connects to the C-terminal region (see schematic in Fig. 1A).

The linker helix was only observed in the poised PIC (Abril-Garrido et al., 2023), not other fully-engaged PIC structures.

Thanks for clarifying. We note that some structures of TFIIH alone also see the long helix. Accordingly, we modified this section to read:

“In many TFIIH and PIC structures the linker is not visible, presumably due to flexibility. However, when it is seen (Abril-Garrido et al., 2023; Greber et al., 2019), the linker emerges from the N-terminal domain as a long alpha-helix running along the interface between the two ATPase subunits…”

Page 8 Recent structures (reviewed in (Yu et al., 2023)) show that the Kinase Module would block interactions between the Core Module and other NER factors. Therefore, TFIIH either enters into the NER complex as free Core Module, or the Kinase Module must dissociate soon after.

To my knowledge, this is still controversial in the NER field. I note the potential function on the kinase module is likely attributed to the N-terminal region of Tfb3 through its binding to Rad3.

We are not experts on NER, but in reviews of the field this appears to be a widely held assumption. A 2008 paper from the Egly lab (Coin et al., DOI 10.1016/j.molcel.2008.04.024) is usually cited, which shows that the interaction between XPD (metazoan Rad3) and XPA is likely incompatible with XPD-MAT1 interaction. In addition to the Yu 2023 review, we now also cite a more recent publication that more extensively reviews the models for core TFIIH interactions (van Sluis et al, 2025). We looked at the multiple recently published structures of various TCR-NER and GG-NER intermediate complexes, and none of them show the CAK module or even the Tfb3/Mat1 N-term, even though those proteins were typically included during assembly. We also consulted with our colleagues Johannes Walter and Lucas Farnung, who are studying various TC-NER intermediates biochemically and structurally. Although the CAK module is included in their assembly reactions, it is not visible in their cryoEM structures. They tell me that the presence of CAK would be compatible with early TC-NER intermediates, but is predicted to overlap with later interactions of XPD with the TC-NER factor STK19 (see Mevissen et al., Cell 2024). To be conservative, we modified the sentence to say “Recent structures … suggest” rather than “show”.

Because the yeast strains used in Fig. 6 retain the N-terminal region of Tfb3, the UV sensitivity assay presented here is unlikely to directly address the contribution of the kinase module to NER.

We agree that our experiment only shows that the connection between Tfb3 N- and C-term domains is not necessary for NER. The individual domains might still be able to function independently. Accordingly, we changed the heading of that section from “Disconnected core TFIIH does not cause an NER defect” to “Split Tfb3 does not cause an NER defect.” This more closely matches the figure legend title.

Page 11. Notably, release of the Tfb3 Linker contact also results in the long alpha-helix becoming disordered (Abril-Garrido et al., 2023), which could allow the kinase access to a far larger radius of area. This flexibility could help the kinase reach both proximal and distal repeats within the CTD, which can theoretically extend quite far from the RNApII body.

Although the kinase module was resolved at low resolution in all PIC-Mediator structures, these structural studies consistently reveal the same overall positioning of the kinase module on Mediator, indicating that its localization is constrained rather than variable. This observation suggests that the linker region may help position the kinase module at this specific site, likely through direct interactions with the PIC or Mediator. This idea is further supported by numerous cross-links between the linker region and Mediator (Robinson et al., 2016).

That is true. But please note that this sentence was meant to describe movement of the kinase module AFTER release from Mediator (see previous sentence). Re-reading the passage, we realized the confusion is because we propose multiple possible pathways in that paragraph. In the first half, we suggest the capture of the kinase module by Mediator might trigger the conformation changes in the linker. In the second half (where it says “Alternatively….”) we suggest the Mediator-CAK interaction could instead come first, and the release of this contact could free the CAK module to move around. We have modified the paragraph to make it clear these are two different distinct models.

Comments on revisions:

Revised ms clarified all my points, including those I previously misunderstood.

Thanks again for helping us improve the manuscript.

**Reviewer #2 (Public review):**

Summary:

This work advances our understanding of how TFIIH coordinates DNA melting and CTD phosphorylation during transcription initiation. The finding that untethered kinase activity becomes "unfocused," phosphorylating the CTD at ser5 throughout the coding sequence rather than being promoter-restricted, suggests that the TFIIH Core-Kinase linkage not only targets the kinase to promoters but also constrains its activity in a spatial and temporal manner.

Strengths:

The experiments presented are straightforward and the model for coupling initiation and CTD phosphorylation and for evolution of these linked processes are interesting and novel. The results have important implications for the regulation of initiation and CTD phosphorylation.

Comments on revisions:

The revised version with revisions to figures, text and new data has addressed all of our prior comments.

We thank the reviewer for helping us improve the paper.

**Reviewer #3 (Public review):**

Summary:

Eukaryotic gene transcription requires a large assemblage of protein complexes that govern the molecular events required for RNA Polymerase II to produce mRNAs. One of these complexes, TFIIH, comprises two modules, one of which promotes DNA unwinding at promoters, while the other contains a kinase (Kin28 in yeast) that phosphorylates the repeated motif at the C-terminal domain (CTD) of the largest subunit of Pol II. Kin28 phosphorylation of Ser5 in the YSPTSPS motif of the CTD is normally highly localized at promoter regions, and marks the beginning of a cycle of phosphorylation events and accompanying protein association with the CTD during the transition from initiation to elongation.

The two modules of TFIIH are linked by Tfb3. Tfb3 consists of two globular regions, an N-terminal domain that contacts the Core module of TFIIH and a C-terminal domain that contacts the kinase module, connected by a linker. In this paper, Giordano et al. test the role of Tfb3 as a connector between the two modules of TFIIH in yeast. They show that while no or very slow growth occurs if only the C-terminal or N-terminal region of Tfb3 is present, near normal growth is observed when the two unlinked regions are expressed. Consistent with this result, the separate domains are shown to interact with the two distinct TFIIH modules. ChIP experiments show that the Core module of TFIIH maintains its localization at gene promoters when the Tfb3 domains are separated, while localization of the kinase module, and of Ser5 phosphorylation on the CTD of Pol II, is disrupted. Finally, the authors examine the effect of separating the Tfb3 domains on another function of TFIIH, namely nucleotide excision repair, and find little or no effect when only the N-terminal region of Tfb3 or the two unlinked domains are present.

Strengths:

Experiments involving expression of Tfb3 domains in yeast are well-controlled and the data regarding viability, interaction of the separate Tfb3 domains with TFIIH modules, genome-wide localization of the TFIIH modules and of phosphorylated Ser5 CTDs, and of effects on NER, are convincing. The experiments are consistent with current models of TFIIH structure and function and support a model in which Tfb3 tethers the kinase module of TFIIH close to initiation sites to prevent its promiscuous action on elongating Pol II.

We appreciate that the reviewer finds that our main conclusions are convincing.

Weaknesses:

The work is limited in scope and does not provide major insights into the mechanism of transcription. The main addition to current models of transcription is that tethering of Kin28 to Tfb3 may limit kinase action from occurring downstream from the initiation site.

The first described experiment, which purports to show that three kinases cannot function in place of Kin28 when tethered (by fusion) to Tfb3 is missing the crucial control of showing that Kin28 can support viability in the same context. This result also does not connect with the rest of the manuscript, although the experiment apparently motivated the subsequent studies reported here.

We elected not to do this control experiment for several reasons. As reviewer 3 points out, this kinase fusion experiment turned out to be somewhat disconnected from the rest of the paper. Even though it didn’t work, we included it in the paper because the results led us to the realization that the Tfb3 C-term was actually not fully essential for viability as reported, which in turn led us to the idea of splitting Tfb3. Structural studies (https://doi.org/10.1126/sciadv.abd4420, https://doi.org/10.1073/pnas.2009627117, https://doi.org/10.7554/eLife.44771) show that, in addition to providing linkage to the core module, the C-term of Tfb3 induces a conformation change in Kin28/Cdk7 necessary for full kinase activity (which is likely why the strains without C-term are just barely viable). If we were to pursue why the fusions didn’t work, we could tether Kin28 directly to the Tfb3 linker (and may try this in the future), but then would need to also express the C-term separately for its activating function. Even then, this would be an imperfect control for the fusion experiments in Figure 1. Because were trying to best mimic Kin28 being tethered via the accessory subunit Tfb3/Mat1, in the Figure 1 experiment we did not directly attach the kinases to Tfb3. For Ctk1/Cdk12, we fused the Tfb3 linker to the Ctk3 accessory subunit (analogous to Tfb3), and for Bur1/Cdk9, we fused to the cyclin subunit Bur2 (there is no known third subunit in this complex). The one exception was Mpk1, which has no partner subunits and is not a CDK. There are many reasons why this high-risk protein fusion experiment may not have worked, but chose not to pursue it further at this time.

Finally, the authors present the interesting and reasonable speculation that the TFIIH complex and connecting Tfb3 found in mammals and yeast may have evolved from an earlier state in which the two TFIIH subdomains were present as unconnected, distinct enzymes. It will be interesting to have this idea tested more thoroughly as more molecular evolutionary data becomes available.

Comments on revisions:

For the most part, the authors have satisfactorily addressed my previous critique. In particular, they have added to their discussion of evolutionary implications, and performed an experiment casting doubt on the assertion of a dominant negative effect, and as a consequence removed this claim from the manuscript. I also pointed out that the fusion experiments that lead off the Results section are missing the crucial control of including a Tfb3-Kin28 fusion. The authors have elected not to perform this control experiment, pointing out that even this control would be imperfect in some respects, and agreeing that this experiment is somewhat disconnected from the rest of the paper. The reason for including it, in spite of its somewhat tangential nature, is that it provides something of a rationale for the experiments that follow. I don't so much mind their retaining the experiment, as the absence of this control (and indeed, the results) does not so much impact the later results. However, I think if it is to be included, this shortcoming should be explicitly recognized, especially as a service to younger scientists who could benefit from an exposition that includes a thorough consideration of potential control experimenents.

We thank the reviewer for helping us improve the paper.
